# The Landscape of Signaling Pathways and Proteasome Inhibitors Combinations in Multiple Myeloma

**DOI:** 10.3390/cancers13061235

**Published:** 2021-03-11

**Authors:** Tina Paradzik, Cecilia Bandini, Elisabetta Mereu, Maria Labrador, Elisa Taiana, Nicola Amodio, Antonino Neri, Roberto Piva

**Affiliations:** 1 Department of Molecular Biotechnology and Health Sciences, University of Torino, 10126 Torino, Italy; tina.paradzik@unito.it (T.P.); cecilia.bandini@unito.it (C.B.); elisabetta.mereu@unito.it (E.M.); maria.labradorgranados@unito.it (M.L.); 2 Department of Oncology and Hemato-oncology, University of Milano, 20122 Milano, Italy; elisa.taiana@unimi.it (E.T.); antonino.neri@unimi.it (A.N.); 3 Hematology Unit, Fondazione Cà Granda IRCCS, Ospedale Maggiore Policlinico, 20122 Milano, Italy; 4 Department of Experimental and Clinical Medicine, Magna Graecia University of Catanzaro, 88100 Catanzaro, Italy; amodio@unicz.it; 5 Città Della Salute e della Scienza Hospital, 10126 Torino, Italy

**Keywords:** multiple myeloma, proteasome inhibitors, drug resistance, combinatorial treatment, synthetic lethality

## Abstract

**Simple Summary:**

In the last decade, proteasome inhibitors (PIs) have become a standard for the treatment of multiple myeloma (MM). As a consequence of the pleiotropic effects of PIs on various signaling pathways, synergistic or additive activities with other anti-myeloma therapies have been identified and approved for clinical use. However, the complex biology of the MM disease inevitably triggers resistance also to combined regimens. Complex loops within cellular pathways, crosstalk with the bone marrow microenvironment, and considerable toxicities are accountable for the poor responses of new multidrug treatments. High-throughput functional approaches are allowing the identification of a multitude of previously undescribed synthetic lethal interactions. In the present review, we explore recent investigations on novel combination strategies that could overcome drug resistance and broaden the applicability of PIs to other hematological malignancies and solid tumors.

**Abstract:**

Multiple myeloma is a malignancy of terminally differentiated plasma cells, characterized by an extreme genetic heterogeneity that poses great challenges for its successful treatment. Due to antibody overproduction, MM cells depend on the precise regulation of the protein degradation systems. Despite the success of PIs in MM treatment, resistance and adverse toxic effects such as peripheral neuropathy and cardiotoxicity could arise. To this end, the use of rational combinatorial treatments might allow lowering the dose of inhibitors and therefore, minimize their side-effects. Even though the suppression of different cellular pathways in combination with proteasome inhibitors have shown remarkable anti-myeloma activities in preclinical models, many of these promising combinations often failed in clinical trials. Substantial progress has been made by the simultaneous targeting of proteasome and different aspects of MM-associated immune dysfunctions. Moreover, targeting deranged metabolic hubs could represent a new avenue to identify effective therapeutic combinations with PIs. Finally, epigenetic drugs targeting either DNA methylation, histone modifiers/readers, or chromatin remodelers are showing pleiotropic anti-myeloma effects alone and in combination with PIs. We envisage that the positive outcome of patients will probably depend on the availability of more effective drug combinations and treatment of early MM stages. Therefore, the identification of sensitive targets and aberrant signaling pathways is instrumental for the development of new personalized therapies for MM patients.

## 1. Introduction

### 1.1. Multiple Myeloma

Multiple myeloma (MM) is a cancer of terminally differentiated plasma cells and represents around 10% of diagnosed hematological malignancies in developed countries [1]. It is characterized by the expansion of clones carrying one or more genetic alterations within the bone marrow [2]. Although MM is a genetically heterogeneous disease [3], a common feature of malignant plasma cells is the production of abnormally large amounts of immunoglobulins, which can be detected in the blood and urine of patients [1]. The accumulation of antibodies causes organ dysfunctions revealed by hypercalcemia, renal insufficiency, anemia, and bone lesions (known as the CRAB criteria), that marks the existence of the symptomatic disease [4]. Genetic complexity poses a great challenge to find effective therapies for MM that, despite great improvements during the last decade, remains an incurable disease.

In recent years, different large-scale analyses [3,5,6] pinpointed the importance of chromothripsis (a single catastrophic event leading to localized chromosomal rearrangements) and hyperdiploidy for the early evolution of the disease from monoclonal gammopathy of undetermined significance (MGUS) to smoldering multiple myeloma (SMM). Next, events such as copy number variations and the emergence of single-nucleotide polymorphisms were recognized as drivers of disease progression. Additional alterations, including aberrant DNA methylation and microRNA (miRNA) expression, are thought to contribute to the development of more advanced MM stages [1]. Finally, the interplay with the bone microenvironment has been shown to play a significant role in myeloma pathogenesis [1,7].

### 1.2. Advances in Multiple Myeloma Treatment Using Proteasome Inhibitors

**Figure 1:**
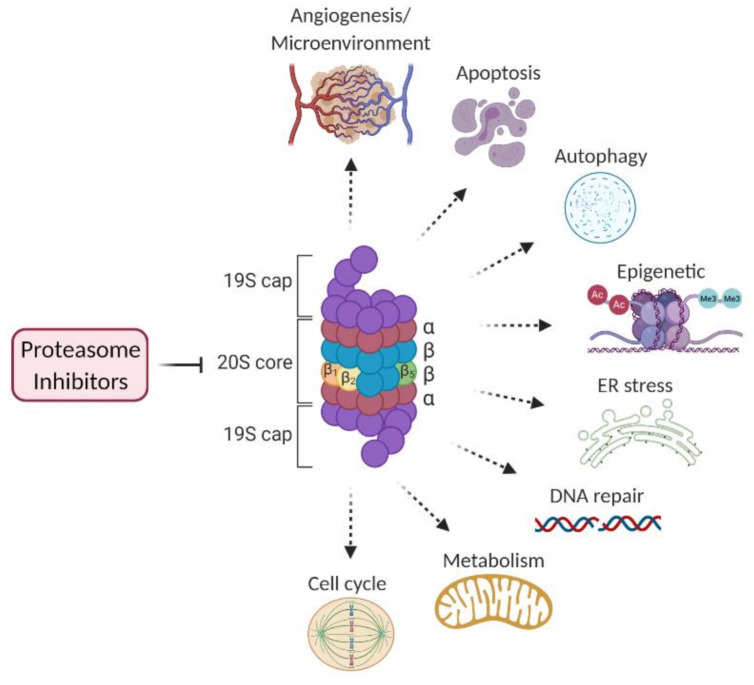
Cellular processes affected by proteasome inhibition. Druggable targets within these pathways are a reservoir of synthetic lethal partners to proteasome inhibitors (PI).

The ubiquitin–proteasome system (UPS) and the autophagy–lysosome system represent two crucial types of machinery for protein degradation. While levels of autophagy mostly depend on the growth conditions, the UPS is constantly mediating protein turnover to regulate various cellular functions, including cell cycle, cell survival, apoptosis, cellular metabolism, and protein quality control [8]. This system has to be tightly regulated to maintain homeostasis. Since plasma cells produce high amounts of immunoglobulins, they are very sensitive to the deregulation of proteindegradation. Malignant plasma cells are even more susceptible to proteasomal inhibition than normal plasma cells. Among other factors, this can be attributed to the constitutive activation of the NF-κB signaling pathway in MM [9,10]. NF-κB plays a key role in the regulation of many targets which tumor growth depends on. Proteasome inhibitors (PI) block IκB degradation and thus, indirectly, inhibit NF-κB signaling [2]. However, other processes that contribute to the antitumor effects of PIs include inhibition of altered cell cycle control and apoptosis [11,12], endoplasmic reticulum stress [13], angiogenesis [14], and DNA repair [15] (Figure 1). The sensitivity of malignant cells to PIs and the design of successful clinical protocols have led to the approval of PIs to treat multiple myeloma, and today three PIs are routinely used in clinics [2,16]. The first-in-class PI was bortezomib, a slowly reversible inhibitor of the β5 catalytic proteasomal subunit. Next, the irreversible inhibitor of β5 site carfilzomib, and the first orally administered PI ixazomib were approved [2]. Among developing PIs, marizomib has the distinctive property to inhibit multiple catalytic sites within the 20S core of proteasome [17].

Although the advent of PIs has highly improved the clinical outcome of MM, there is a significant proportion of patients who relapse or are intrinsically resistant to this class of drugs. Resistance can arise through many cellular responses mediated by the downstream effects of proteasomal inhibition [2]. However, as a consequence of the pleiotropic effects of PI’s, synergistic or additive activities with other antimyeloma therapies have been identified [2]. At present, approved combinations for clinical use include dexamethasone and immunomodulatory drugs (lenalidomide), chemotherapy (doxorubicin, mephalan, or cyclophosphamide), antibodies (elotuzumab or daratumumab), or histone deacetylase (HDAC) inhibitors (panobinostat) [18]. Such multiple drug combinations have become the standard initial approach in MM, specifically in patients ineligible for transplantation [1,19].

Along with high-dose therapy and autologous hemopoietic stem cell transplantation, the use of drug combinations targeting different pathways in MM has led to better clinical responses. In the future, the positive outcome of patients will probably depend on the availability of more effective drug combinations and the treatment of early MM stages [1]. Therefore, the identification of sensitive targets and aberrant signaling pathways is instrumental for the development of new personalized therapies for MM patients [20].

Although the toxicity of PIs is well controlled in clinics, distinct adverse profiles (such as peripheral neuropathy and cardiotoxicity) frequently arise [21]. To this end, the use of combinatorial treatments might allow lowering the dose of inhibitors and therefore, minimize their side-effects. In addition, although PIs failed as single agents for the treatment of solid tumors in clinics [16], there are accumulating pieces of evidence that the combination of proteasomal inhibition with various drugs could improve the outcome of a wide range of malignant diseases [22,23,24,25].

In the present review, we explore recent investigations on novel combination strategies that could overcome drug resistance and broaden the applicability of this class of drugs to other hematological malignancies and solid tumors.

## 2. Approaches Allowing the Discovery of New Effective Drug Combinations

Synergy is identified when two compounds increase each other’s effectiveness by more than the sum of their single-agent responses. Methods to quantify drug synergies are based on old probabilistic theories, described elsewhere in detail [26,27,28]. Each of these models has its associated limitations, and yet there is no agreement regarding the appropriate methods for synergy quantification [29,30,31,32,33]. Defining a consensus model is not trivial since different methods applied to the same data may give divergent results [34,35]. The impact of this division hampers reproducibility between studies, delays progress in the discovery of truly synergistic drug combinations, and negatively impacts the translation of combination discovery efforts into the clinics. Combinatorial treatments have significant advantages over therapies with single drugs: first, toxicity reduction by minimizing doses; second, outcomes improvement by an escalating effect. In the context of drug resistance, attacking multiple targets may reduce or delay the development of resistance.

Multiagent therapy is the cornerstone of treatment in multiple myeloma. Recent advances in clinical outcomes for MM patients are derived from the combination of novel agents, such as dexamethasone and immunomodulatory drugs, (lenalidomide), chemotherapy (doxorubicin, mephalan, or cyclophosphamide), antibodies (elotuzumab or daratumumab), or histone deacetylase (HDAC) inhibitors [18,36]. The common rationale is to address the clonal heterogeneity of the MM, thus combining different agents to eradicate both dominant and minor tumor clones. Re-emergence of resistant clones can occur during relapse, supporting the idea of a combinatorial approach in which one drug re-sensitize the cells to the original treatment to revert innate or acquired resistance [37].

Combinatorial screenings as well as predicting algorithms are widely used for the identification of synergistic drug combinations. Functional genetic screenings represent a powerful tool for discovering combinatorial treatment by detecting drug sensitizers with high accuracy, thereby guiding the development of new strategies capable of overcoming drug resistance [38]. Several studies in MM used siRNA [39,40], shRNA [41,42,43] or CRISPR/Cas9 [44,45,46,47] libraries to discover synthetic lethal interactions that potentiate the therapeutic effects of a given drug.

However, loss-of functions perturbations using RNAi and CRISPR also show certain limitations. Potential complications and artefacts, when RNAi is used to identify target, include incomplete target knockdown and off-target degradation of unintended mRNA transcripts [48,49]. By contrast, CRISPR-Cas9 technology can lead to the induction of the DNA damage causing cell cycle stalling and cell death, exon skipping or alternative splicing, which may result in the translation of functional truncated protein, and genetic compensation, leading to a partial rescue phenotype [49,50]. Thus, genetic perturbation screenings may result in incorrect identification of target genes; however, the use of complementary orthogonal approaches can minimize the likelihood that one technique’s shortcomings lead to false-negative or false-positive findings [49,51].

With reference to proteasome inhibitors, Zhu at al. identified 37 genes that enhance bortezomib activity in the multiple myeloma cells upon silencing. Among these, cyclin-dependent kinase 5 (CDK5) was one of the most potent sensitizer. The observed synergy was further confirmed using specific of CDK5 inhibitors [39]. More recently, Bergaggio et al. identified isocitrate dehydrogenase 2 (IDH2) as a druggable target whose inhibition sensitizes multiple myeloma, mantle cell lymphoma, and Burkitt lymphoma cells to proteasome inhibitors [42]. A genome-wide CRISPR/Cas9 screening identified the proteasome regulatory subunit (PSMC6) as a top gene conferring bortezomib resistance in human multiple myeloma cells. Concordantly, gene deletions or down regulation of 19S proteasome subunit expression were described in PI-resistant patients [46]. By combining in silico analysis and CRISPR/Cas9 library screenings, Xie H. et al. identified SENP2 (Sentrin/SUMO-specific proteases-2) as a bortezomib sensitive gene and found its expression downregulated in bortezomib resistant MM patients. Furthermore, SENP2 down regulation potentiates bortezomib resistance development by activating NF-κB pathway, whereas overexpression of SENP2 sensitized the cells to bortezomib treatment [44].

Thus, strategies based on functional genetic screenings have the potential to unbiasedly identify clinically actionable drug combinations that can prevent or overcome drug resistance. However, as genetic perturbations are profoundly different from biochemical inhibition, the relationship between synthetic lethality and drug synergy is likely case-dependent [52]. In light of this, high-throughput drug screenings can accelerate the discovery of synergistic drug combinations. Recently, Bonolo de Campos [53] assembled a standardized MM drug panel and screening platform for drug profiling in 25 MM cell lines, 15 non-Hodgkin’s lymphoma cell lines, and in 113 primary MM samples. This study identified subpopulations of patients with distinct drug sensitivity patterns linked to genetic and mutational profiles, and clinical outcomes. These patterns highlighted vulnerabilities that can be exploited for functional studies and combination therapy development [53].

Although high-throughput screenings have been successfully implemented, it is still impractical to test all the possible drug combinations, even for a reduced subset of anticancer drugs. Thus, several computational methods for synergy prediction have recently been developed [54,55,56,57]. Recently, a study using simulated treatment learned signatures (STLsig), a machine learning method to identify predictive gene expression signatures, was performed [58]. Such approaches could aid patients and provide insights into the biological mechanism behind treatment benefits. However, because predicting algorithms depend on the structure of the training dataset and the protocol of drug synergy calculation, they cannot be reproducibly adapted to different studies [52]. Therefore, a consensus on the protocols to measure drug synergies is urgently needed to improve the power of prediction of machine learning algorithms.

## 3. Emerging Proteasome Inhibitors Drug Combinations Targeting Different Molecular Pathways

### 3.1. Immunotherapy

The immunomodulatory drugs (IMiDs) thalidomide and its derivatives lenalidomide and pomalidomide are key treatment modalities for hematologic malignancies, including MM. Although in use for a long time, the mechanism of IMiDs activity has been revealed during the last decade [21,59,60]. Cereblon (CRBN), which belongs to an E3 ubiquitin ligase complex was identified as the primary target of IMiDs [61]. This is also supported by the fact that MM cell lines lacking CRBN are highly resistant to IMIDs [62]. The complex of cereblon, damaged DNA binding protein 1 (DDB1), Cullin-4A (CUL4A), and regulator of cullins 1 (ROC1) operates by ubiquitinating several proteins. Binding of IMiDs alters the substrate specificity of CRBN, leading to the recruitment and degradation of IKZF1 and IKZF3, and consequent downregulation of proteins that regulate tumor proliferation and survival such as IRF4 and MYC. However, many IMiD activities can be attributed to ubiquitin-independent chaperone-like mechanisms of action [59]. The success of combinatorial treatment with PIs can be ascribed to the inhibition of both ubiquitin- and ubiquitin-independent pathways.

The combination of PIs with IMiDs is currently one of the most effective approaches in MM patients [1]. Significantly, a phase III trial (NCT00644228) demonstrated the improvement of progression-free survival and overall survival in newly diagnosed patients [63]. Consequently, bortezomib + lenalidomide + dexamethasone (VRD) is considered standard initial treatment for all MM patients who can tolerate multi-drug combinations [1]. Several other combinations of PI and immunomodulatory agents have already been evaluated or are in clinical trials (Table 1). Of note, the combination of bortezomib with the first generation IMiD thalidomide turned out to have more side-effects, with a high occurrence of peripheral neuropathy as compared to the lenalidomide-bortezomib combination [21]. Unfortunately, MM patients who became refractory to these agents experienced significantly worse outcomes [64].

Various combinations of monoclonal antibodies with PIs have been recently reviewed [85,86]. When used as monotherapy, monoclonal antibodies infrequently produce a significant response in MM patients, thus requiring combination with other agents [86]. Several relevant targets demonstrated no therapeutic activity in multiple myeloma. The anti Il-6 antibody (siltuximab) did not display advantages as a single agent nor in combination with bortezomib [87]. Likewise, the first generation anti-SLAMF7/CD319 antibody elotuzumab did not show efficacy as monotherapy [86]. SLAMF7/CD319 is a receptor highly expressed in MM cells [88]. It mediated the activation of NK cells and the inhibition of MM cell adhesion to bone marrow. As a consequence of encouraging clinical results in combinations with lenalidomide/dexamethasone, elotuzumab was approved for the treatment of patients who received several prior lines of therapy [85]. Combinations of this antibody with carfilzomib and dexamethasone are under phase II clinical study (Table 1).

The anti-CD38 antibody daratumumab was approved in 2017 for the treatment of MM in combination with bortezomib and dexamethasone [89], based on excellent progression-free survival and overall rate response (ORR) from the CASTOR trial [90]. Recently, the results from phase-3 study CANDOR study were published, showing significant progression- free survival and favorable benefit–risk profile of carfilzomib, daratumumab and dexamethasone combination [66]. It is known that daratumumab antimyeloma effects occur via multiple mechanisms of action by acting both on MM cells and components of the immune system, such as immunosuppressive CD38+ T regulatory cells [91]. The exact mechanism of synergy with PI is still unclear; however, it can be ascribed to the pleiotropic activity of PI on both MM cells and the microenvironment [92]. A second anti-CD38 monoclonal antibody, isatuximab, has been recently approved in combination with pomalidomide and dexamethasone for the treatment of RRMM patients based on ICARIA-MM trial (NCT02990338) [93]. Isatuximab combinations with bortezomib, carfilzomib, and other drugs are currently under clinical evaluation (Table 1).

Preclinical studies evaluating proteasome targeting in combination with different aspects of the immune system are listed in Table 2. Immune checkpoints represent critical pathways that serve to modulate immunological responses and self-tolerance [94]. These pathways are often exploited by tumors to evade the immunological system. The immune checkpoint CD47, also known as the “do not eat me” signal, was found overexpressed in MM cells and positively correlated with the stage of disease [95] TTI-622 (SIRPα-IgG4 Fc), is a soluble recombinant fusion protein created by directly linking the sequences encoding the N-terminal CD47 binding domain of SIRPα with the Fc domain of human immunoglobulin (IgG4) [95]. It was shown that TTI-622 efficiently binds CD47 and prevents delivering inhibitory signals to macrophages. A trial of TTI-622 in combination with various drugs, including carfilzomib, is ongoing in RRMM patients (NCT03530683).

Another promising approach in MM is the use of oncolytic viruses. Specifically, the reovirus receptor JAM-A was found overexpressed in several MM cell lines and primary samples from patients [129]. Recently, Solimando et al. [130] have demonstrated that JAM-A could be a prognostic factor in MM, since group of patients exhibiting lower expression of JAM-A had significantly longer progression-free survival and overall survival. Phase I clinical data suggested that reovirus treatment (Reolysin) could be effective when associated with other drugs [131]. An ongoing trial is evaluating the combination of carfilzomib and reovirus (NCT02101944). There is evidence that PIs enhance reovirus entry, infection, and killing of MM through the improvement of early innate response by CD14+ cells (monocytes) [132]. Additionally, it was reported that PIs induce direct T-cell activation and potentiate T-cell killing activity against reovirus infected MM cells. Interestingly, carfilzomib/reovirus combination was found to significantly increase PD-L1 expression in MM cells of patients with clinical response to protocol therapy. This observation led to a clinical trial (NCT03605719) aimed to assess the effects of the immune checkpoint PD-1/PD-L1 inhibitor nivolumab to the above-described combination (carfilzomib/reovirus) [133]. Overall, immunotherapeutic approaches are becoming an essential component of MM management and lots of efforts have been made to the development of new therapies aimed to tackle MM-associated immune dysfunction [86]. As a matter of fact, combinations of PIs and different immunotherapies are finding their way into the clinics, showing superior results in the combinatorial treatments of MM.

### 3.2. Targeting the MM-Microenvironment Crosstalk with Tyrosine Kinase Inhibitors and Proteasome Inhibitors

#### 3.2.1. Receptor Tyrosine Kinases

Receptor tyrosine kinases (RTKs) represent the starting point of most cellular signaling pathways. One of the best scientific rationales to target RTKs in MM stems from vascular endothelial growth factor (VEGF) studies. VEGF is an important regulator of angiogenesis, cell migration, survival, and drug resistance [134]. Since angiogenesis was demonstrated to play a critical role in MM, VEGF and its receptor were considered as promising therapeutic targets in MM. Various VEGF inhibitors showed favorable activities in preclinical studies. However, these data have not been confirmed in clinical trials. It was proposed that the efficacy of angiogenesis inhibitors could be exploited in combination with other drugs. Sorafenib, a multi-kinase inhibitor that acts mostly through inhibition of RAF-kinase and VEGF receptor 2, showed a synergistic effect in combination bortezomib [135]. Indeed, sorafenib completely abrogated MCL-1 upregulation induced by IL-6 and VEGF in myeloma cells. However, paradoxical upregulation of AKT phosphorylation and decreased phosphorylation of the STAT3 and MEK/ERK were detected. To date, there are no significant conclusions from clinical trial inspecting sorafenib/PI combination (NCT00303797).

The c-MET receptor was shown to be highly expressed in MM cells and in the bone microenvironment. Several MET inhibitors were tested in clinical trials with limited success [134]. Despite preclinical data on the efficacy of the MET inhibitor cabozantinib in combination with bortezomib are insufficient, and that animals used in these studies have suffered from severe side effects [136], a combination of cabozantinib and bortezomib is currently under clinical trial (Table 1).

The insulin growth factor type 1 (IGF-1) pathway was found upregulated in bortezomib-resistant MM cell lines, as a consequence of increased IGF-1 secretion and IGF-1R activation [137]. IGF-1R is a tyrosine kinase that can be activated also by insulin and its expression correlates with poor survival in MM [134,137]. Administration of exogenous IGF-1 reduced bortezomib sensitivity in MM cells. This effect was more pronounced in bortezomib resistant cells. IGF-1R knockdown by shRNA or its pharmacological inhibition by OSI-906 re-sensitized MM cell lines and patient samples resistant to bortezomib. Most importantly, this effect was durable in in vivo models of MM [137].

#### 3.2.2. Non-Receptor Tyrosine Kinases

Bruton’s tyrosine kinase (BTK) is a non-receptor tyrosine kinase that plays a crucial role in the proliferation and survival of malignant B cells and their interactions with the tumor microenvironment [134]. It is a key player downstream of the B-cell receptor (BCR), which is also involved in chemokine, Toll-like (TLR), and Fc receptors signaling [138]. BTK was often found overexpressed in MM where its activation in the bone marrow microenvironment promotes MM cell growth, survival, interaction with other stromal components, and MM-induced bone lysis [139]. A study using the BTK inhibitor CC-292 in combination with carfilzomib has shown antimyeloma activity with a positive impact on the bone microenvironment due to decreased osteoclasts function [96]. It is known that proteasome inhibition in MM downregulates BTK via NF-κB signaling pathway [140]. However, BTK was found overexpressed in PI-resistant MM, possibly as a consequence of constitutive NF-κB activation [141]. Interestingly, MM cells could be re-sensitized to bortezomib by BTK RNA interference or treatment with the irreversible BTK inhibitor ibrutinib. Notably, naïve nor resistant cells, as well as naïve and relapsed primary cells, were particularly sensitive to ibrutinib alone [142]. Currently, ibrutinib combinations with bortezomib or carfilzomib are under clinical evaluation for the treatment of RRMM patients (Table 1). A phase I trial demonstrated promising results with 67% ORR [69].

The Janus kinase (JAK)–signal transducer and activator of transcription (STAT) pathway regulates cell proliferation, differentiation, migration, and apoptosis, being strongly interconnected with other signaling pathways [143]. The JAK/STAT pathway was frequently found activated by IL-6 in MM and thus considered a suitable therapeutic target. A trial evaluating the combination of the JAK1/2 inhibitor ruxolitinib with carfilzomib and dexamethasone (NCT03773107) is currently in the recruitment phase.

In summary, tyrosine kinase receptors have been studied for a long time as a potential targets for MM treatment. Combinations with PIs had also shown promising in vitro activities. However, most of clinical studies did not confirmed these observations. Since BTK inhibitor ibrutinib/PI combination showed good results in phase I, it would be interesting to see whether this will be confirmed in subsequent phases of clinical trials.

### 3.3. Targeting PI3K/AKT/mTOR Pathway

**Figure 2:**
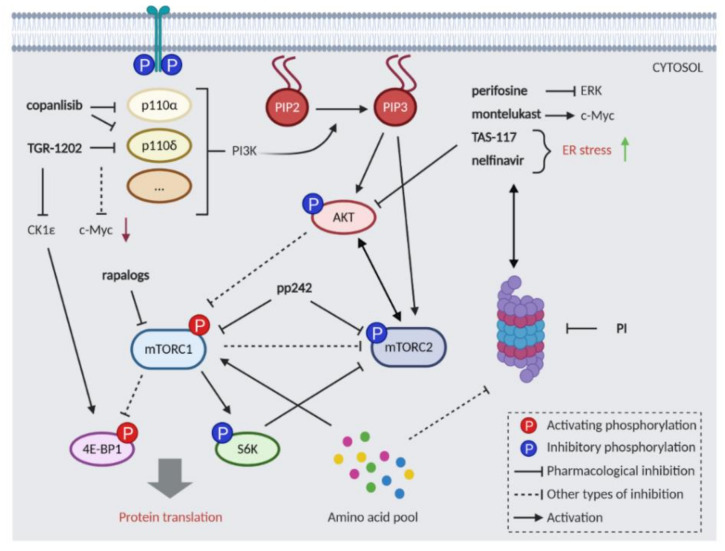
Inhibition of proteasome inhibitor (PI) and PI3K/AKT/mTOR pathway results in synergistic cellular death. The PI3K/AKT/mTOR pathway is a central signaling hub in eukaryotic cells and it is connected to the ubiquitin–proteasome system (UPS) by balancing amino acid homeostasis (amino acid pool). Intensive feedback loops between mTORC1, mTORC2, and AKT pose a challenge to the successful inhibition of this pathway. Rapalogs target the mTORC1 complex; the mTORC1/mTORC2 dual inhibitor pp242 (torkinib) is more effective in combination with PI. The PI3K inhibitors copanlisib and TGR-1202 demonstrated synergistic cytotoxicity with PI. Several AKT inhibitors (perifosine, TAS-117, nelfinavir, montelukast) were found to synergize with (PI) by affecting endoplasmic reticulum (ER) stress, ERK, or c-Myc.

The PI3K/AKT/mTOR pathway represents a major eukaryotic signaling network involved in the regulation of physiological responses to external stimuli [144]. Signals from growth factors, cytokines, and other molecules are integrated by phosphatidylinositol 3-Kinases (PI3Ks), as reviewed in [145]. Activated PI3Ks recruit to the cell membrane the serine-threonine kinase AKT, which can phosphorylate multiple downstream targets, including mTOR (Figure 2) [146]. This pathway has a key role in the control of the synthesis of new cellular components, including proteins, through an amino-acid sensing system [8,147]. On the other hand, it suppresses catabolic processes, such as autophagy and inhibits apoptosis [147]. Although activating mutations are present in a very low percentage of patients, the PI3K/AKT/mTOR pathway plays a critical role in MM cell growth. Molecules secreted in the microenvironment by tumor and stromal cells, such as IL-6, are responsible for the activation of the mTOR pathway [148]. It is known that mTOR inhibition leads to activation of the protein degradation machinery. Therefore, the simultaneous targeting of these two systems could be a rational approach to induce MM cell death (Figure 2). Several drugs developed to target PI3Ks or other components of the pathway have been tested in combination with PIs (Table 2).

PI3Ks are recruited to the membrane upon receptor activation and transmit the signals to downstream targets by phosphorylation of phosphatidylinositides (PtdIns). PI3Ks are grouped into three classes (IA, IB, II and III) based on their structures and substrate specificity [145]. A recent study combining bortezomib and copanlisib, a dual PI3Kα and δ inhibitor (both class IA), demonstrated synergistic cytotoxicity in MM cell lines and primary patient samples [100]. It was shown that PI3K inhibition strongly impaired c-MYC translation [101]. Accordingly, the PI3Kδ inhibitor TGR-1202 synergized with carfilzomib by silencing c-MYC in different hematological malignancies. However, in this study TGR-1202 also inhibits the activity of casein kinase 1ε (CK1ε), known to directly activate 4E-BP1, a downstream target of mTORC1 [149]. The synergistic effect was rescued by c-MYC or 4E-BP1 overexpression. Interestingly, montelukast, a cysteinyl-leukotriene receptor (CysLTR) antagonist, was shown to act synergistically with carfilzomib through a CysLTR independent pathway. Specifically, the drug combination decreased c-MYC translation through mTOR, possibly via protein synthesis inhibition [99].

The Ser/Thr kinase AKT represents a central hub downstream of PI3Ks [143]. Perifosine, a non-selective inhibitor of AKT phosphorylation, showed a favorable profile when combined with different drugs, as well as radiation [150]. Even though it is known that bortezomib activates AKT, it has been shown that the combination of perifosine with bortezomib blocks AKT and ERK signaling pathways and induces apoptosis in MM models. However, a phase III of clinical trial was terminated due to lack of efficacy [151]. TAS-117, an allosteric inhibitor of AKT, was particularly active in MM cell lines with high basal levels of p-AKT and abrogated the effects of bone marrow stromal cells (BMSCs) on AKT activation [102]. Importantly, the secretion of cytokines from BMSCs was decreased as a consequence of NF-κB inhibition. TAS-117 combinations with bortezomib or carfilzomib were synergistic in MM cells and in xenograft mouse models, independently of AKT activation. TAS-117 also enhanced fatal ER stress induced by proteasome inhibitors in MM [102].

The protease inhibitor nelfinavir, known to inhibit AKT phosphorylation, demonstrated single agent antineoplastic activity in several human cancers, including MM [70]. The combined treatment with PIs induces unfolded protein response (UPR) through IRE1 and XBP1 proteins activity in MM cells in vitro [152]. Ongoing clinical trials including bortezomib and nelfinavir [70] (NCT02188537) had so far shown an ORR of 65% in patients treated with several prior drugs, with a subgroup of triple-refractory patients showing 62% ORR.

The rapamycin derivatives (rapalogs) temsirolimus and everolimus, inhibitors of mTORC1, were first approved for the treatment of advanced renal cell carcinoma. Nevertheless, in clinical trials, rapalogs failed to achieve significant effects as monotherapy [144] and in combination with PIs [72]. The main flaw of rapalogs is their inability to completely block the phosphorylation of all mTORC1 substrates and the consequent feedback activation of AKT (Figure 2). Inhibitors of both mTORC1 and mTORC2 were developed to overcome the activation of AKT. Among these, pp242 (torkinib) was more effective than rapamycin and its combination with bortezomib led to a synergistic anti-MM effect [98]. The authors proposed that the main toxicity of this drug is through mTORC2 inhibition. Contrary to mTORC1, which controls cell growth and metabolism, mTORC2 regulates proliferation, survival, and cytoskeleton primarily by activating several members of the AGC protein kinase family, including PKB/AKT, PKA/PKC/PKG, and SGK1 [147].

Myristoylated alanine-rich C-kinase substrate (MARCKS) is a protein kinase C (PKC) substrate that has been previously reported to play a role in cell adhesion, spreading and mitogenesis. Importantly, MARCKS was found overexpressed in MM cell lines as well as patient samples resistant to bortezomib. Several groups reported synergistic effects of the indirect inhibitor of MARCKS phosphorylation enzastaurin in combination with bortezomib [103,153]. The synergy was confirmed by MARCKS silencing. Phosphorylated MARCKS forms a complex with the transcription factor E21F and binds to the promoter of the SKP2 gene [103]. Thus, MARCKS represents an interesting target downstream of PKC/AKT and the synergistic effects of enzastaurin with PIs could be a good basis for further research.

Despite the rising number of drugs targeting the PI3K/AKT/mTOR signaling pathway (Table 2), few have reached advanced phases of clinical trials raising concerns over dose-limiting toxicities. Complicated connections to other pathways and many feedback loops will require additional efforts to identify the Achille’s heel in PI3K/AKT/mTOR signaling.

### 3.4. Targeting Cell Cycle

Cell-cycle progression is a highly regulated process coordinated by the activation of cyclin–cyclin-dependent kinases (CDKs) and several checkpoint pathways [154]. It is known that the proteasome plays a key role in cell cycle progression [155] by timely degrading ubiquitinated cyclins and checkpoint proteins [156]. Cell-cycle defects are common features in cancer cells. With no exception, deregulation of cyclin D and the INK4 family of CDK inhibitors are key hallmarks of MM. Indeed, one of the most frequent genetic alterations in MM is the t(11;14) translocation, which juxtaposes the immunoglobulin heavy chain (IgH) enhancer to CCND1 gene resulting in aberrant cyclin D1 expression [154]. Although targeting the cell cycle is an attractive therapeutic opportunity in MM, the use of CDK inhibitors is generally limited, since they often induce cytotoxicity in normal cells [146]. A clinical trial using the specific CDK4/6 inhibitor palbociclib in combination with bortezomib did not yield promising results [74]. However, it would be useful to verify whether palbociclib is more suitable for patients with deregulated cyclin D. More recent studies are focused on the inhibition of CDK7, a serine/threonine kinase involved in the regulation of the cell cycle progression, RNA Pol II transcriptional activity, and DNA repair [157] It has been shown that the covalent CDK7 inhibitor THZ1 arrested M cell proliferation in combination with PIs. However, substantial pieces of evidence suggest that THZ1 could also act on other targets, as its effects were not rescued by CDK7 overexpression [104].

The Rho GTPases family member Cdc42 is known to regulate a variety of cellular processes, including cytoskeletal reorganization, cell cycle progression, cell polarity, and transcription [158]. Interestingly, the selective Cdc42 inhibitor CASIN was able to completely sensitize melphalan/bortezomib-resistant MM cells, likely through suppression of STAT3, and ERK transcription factors [105].

Overall, the preclinical results using cell cycle regulators have not been confirmed in clinical studies. Improvements could be achieved through personalized approaches assessing predictive markers, such as t(11;14) translocation. Moreover, since cell-cycle inhibition can result in a selection of non-proliferative clones, novel combination therapies should be designed to target multiple cellular pathways.

### 3.5. Targeting Stress Response and Apoptosis

#### 3.5.1. Targeting Endoplasmic Reticulum Stress

**Figure 3:**
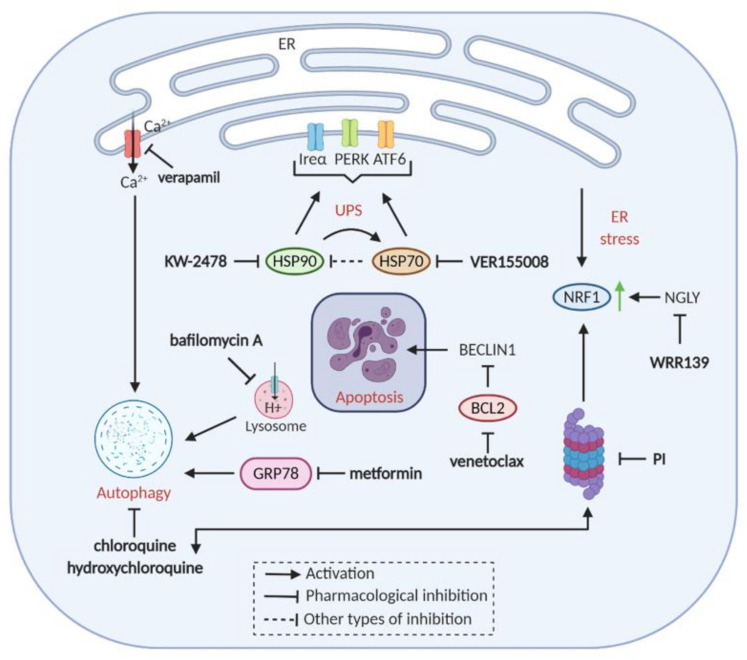
Dual inhibition of cellular stress and proteasome components. Ubiquitin-proteasome system (UPS), endoplasmic reticulum (ER) stress, and autophagy are precisely regulated and connected processes. Simultaneous blockage of targets within these pathways and proteasome leads to apoptosis. Proteasome inhibitors (PI) itself induces ER stress, thus targeting ER stress (verapamil, WRR139, KW-2478, and VER15508) in combination with PI leads to increased cellular death. As compensation to proteasome inhibition, cells turn on autophagy. Drugs affecting autophagy (metformin, bafilomycin A, chloroqouine, hydroxychloroquine) are synthetic lethal with PI. Inhibition of the pro-survival protein BCL2 (venetoclax) leads to increased apoptosis in combination with PI.

Under stress conditions, such as starvation, oxidative stress, or growth factor deprivation, a transient decrease of anabolic processes and cell growth is required. In this scenario, mTORC1 is inhibited resulting in the down modulation of biosynthetic processes and the induction of the ubiquitin-proteasome system (UPS) and autophagy [155]. Since the endoplasmic reticulum (ER) represents the powerhouse of protein synthesis, an important component of the UPS is the endoplasmic reticulum-associated protein degradation (ERAD) [159]. ER stress occurs when the accumulation of unfolded and/or misfolded proteins exceeds the rate of protein refolding or degradation. A series of events followed by the accumulation of unfolded proteins leads to the activation of specific transcription factors (such as ATF4, ATF6, JNK, NRF2 and XPB1) and subsequent induction of various stress-related genes [159]. MM cells are subjected to protein overload due to the massive production of antibodies which causes constant ER stress. It is known that proteasome inhibition also induces ER stress with consequent PERK-eIF2α and IRE1-JNK activation, and thus stimulation of autophagy (Figure 3).

Heat shock proteins (HSP) play a key role in protein homeostasis pathways and in handling the immunoglobulin folding [160]. Since HSPs are involved in many signaling pathways required for MM growth and survival, they represent attractive therapeutic targets. Even though preclinical studies have shown that HSP70 and HSP90 inhibition was synergistic to bortezomib [161,162], antimyeloma activity has not been confirmed in a phase I study [82].

The transcription factor NRF1 represents a unique mechanism by which ER stress regulates UPS. When proteasomal capacity needs to be enhanced or the proteasomal activity is inhibited, the active form of NRF1 is released from the ER and enters the nucleus to upregulate the expression of proteasome subunits [107]. N-glycanase 1 (NGLY) protein was found to have essential role in NRF1 protein activation in response to PI. Inhibition of NGLY protein inhibition inactivates NRF1 and potentiates proteasome inhibitor cytotoxicity in MM and T-ALL (acute lymphoblastic leukemia) cell lines [107] (Figure 3).

#### 3.5.2. Targeting Autophagy

The intensive crosstalk between the proteasome, UPR, and autophagy serves to balance suicidal and protective activities. Upon accumulation of proteasomal substrates (as triggered by PIs), autophagy is activated as a compensatory mechanism [107]. Multiple preclinical studies have demonstrated synergistic toxicity by the simultaneous targeting of proteasomes and autophagy [108,110,163,164,165]. An interesting example is represented by the antidiabetic drug metformin, which was identified as synthetic lethal to PIs in a high-throughput screen aimed to identify drugs that modulated autophagy [110]. The authors demonstrated that the metformin-bortezomib combination delays the growth of myeloma xenotransplants by suppressing GRP78, a key driver of bortezomib-induced autophagy.

ER stress leads to Ca2+ release, which results in the activation of numerous kinases and proteases involved in autophagy [166]. Thus, it was shown that the calcium channel blocker verapamil synergized with bortezomib by enhancing ER stress in MM cell lines [109] (Figure 3).

The autophagy inhibitors chloroquine and hydroxychloroquine, known to impair autophagosome fusion with lysosomes, have shown promising synergistic activities with carfilzomib in vitro and in vivo [163,164,167]. Accordingly, chloroquine/hydroxychloroquine combinations with carfilzomib are in clinical trial for the treatment of RRMM patients (NCT04163107). However, previous phase I/II trials using bortezomib and chloroquine/hydroxychloroquine combinations (NCT01438177, NCT00568880) did not report any significant response [79].

#### 3.5.3. Targeting Apoptosis and DNA Stress

In the last decade, the ER has emerged as a critical structure for apoptosis control in response to a wide variety of stress stimuli. Under acute or sustained ER stress, the UPR actively promotes apoptosis through the upregulation of BCL-2 family pro-apoptotic proteins, increased proteotoxicity, and ROS. The BCL-2 protein family operates as a core to integrate stress signaling networks, regulating cell death, calcium homeostasis, the UPR, and autophagy [166]. The selective inhibition of BCL-2 has proven to be effective in a panel of different cancers by restoring the deranged apoptotic pathway of malignant cells [80]. The BCL-2 inhibitor venetoclax synergized with bortezomib in a preclinical study performed in xenografts co-expressing BCL-2 and MCL-1 proteins [168]. Concordantly, a proportion of MM cell lines and patients resistant to venetoclax treatment showed upregulation of these two proteins [168], and bortezomib was found to neutralize MCL-1 pro-survival activities as a consequence of NOXA induction [12]. Following promising clinical observations [80], a phase III trial (NCT02755597) demonstrated significant efficacy of venetoclax-bortezomib combination in RRMM patients harboring t (11;14) and in tumor cells expressing high levels of BCL-2 [169]. The first results from ongoing trial using carfilzomib, venetoclax and dexamethasone showed overall response rate 100% in t (11;14) patients and 79% in general RRMM patients [81]. However, these analysis were performed on small number of patients. In a more recent clinical study with heavily pre-treated patients the ORR was 37.5%. Strikingly, all responders were t (11;14) positive patients [170].

In addition to its role in protein homeostasis, the ubiquitin-proteasome system is also involved in the regulation of DNA damage repair proteins. It was shown that bortezomib impairs the ability of MM cells to repair DNA double-stranded breaks induced by PARP inhibition [171]. Encouraging preclinical results combining the PARP inhibitor veliparib with bortezomib were followed by a phase I trial in RRMM patients (NCT01495351).

#### 3.5.4. Dual Inhibition of the Ubiquitin-Proteasome System

Even though all PIs available for therapy are designed to target the β5 subunit of the proteasome, several authors have demonstrated that the simultaneous inhibition of β5 and β2 either by combinatorial therapy (bortezomib/carfilzomib and LU-102) or dual inhibition (syringolin analog) can improve the cytotoxic effects of proteasome inhibition [106,172]. Interestingly, the dual inhibitor was able to overcome bortezomib resistance, while the combination of two inhibitors failed to re-sensitize cells. Targeting other components of UPS has also been shown to synergize with PIs. The NEDD8-activating enzyme (NAE) is an essential component of the NEDD8 conjugation pathway that controls the activity of the cullin-RING subtype of ubiquitin ligases (CRLs), thereby regulating the turnover of a subset of proteins upstream of the proteasome [173]. Two NAE inhibitors (MLN4924 and TAS4464) were found to synergize with bortezomib [174,175]. MLN4924 (pevonedistat) combinations with the oral proteasome inhibitor ixazomib are currently in clinical trial for the treatment of RRMM (NCT03770260).

Besides ubiquitin–proteasome system, ER stress management represents another vulnerable spot of MM due to the high production of proteins. Although numerous PI combinations reached clinical evaluation (Table 1), none has confirmed results from preclinical studies and several have shown considerable toxicity. However, due to the great potential of concurrent targeting of these two pathways, different studies are still ongoing and, hopefully, will give a positive contribution to the treatment of MM.

### 3.6. Proteasome Inhibitors and Metabolic Pathways

Cancer cells are typically characterized by altered metabolism; however, due to the high heterogeneity of the disease, the metabolic derangements are extremely diverse. Similar to other cancers, enhancement of glycolysis and glutaminolysis are key features of MM cells [176]. It was also reported that changes in the metabolism induced by hypoxia are instrumental for drug resistance of MM cells [177]. The two main players of hypoxia–associated drug resistance are hypoxia-inducible factor 1α (HIF1α) and lactate dehydrogenase (LDHA). HIF1α is a transcriptional factor regulated by hypoxic microenvironment but also by other factors such as oncogene activation or loss of tumor suppressors [178] Overexpression of HIF1α in the hypoxic environment promotes the upregulation of glucose transporters and glucose enzymes [179]. Importantly, HIF1α metabolic targets, such as Hexokinase 2 (HK2) and LDHA, were overexpressed in plasma cells of relapsed patients. Moreover, specific inhibition of LDHA and HIF1α was able to re-sensitize MM cells to bortezomib in vivo [177].

The first rate-limiting step of glucose metabolism is its transport across the plasma membrane through glucose transporters (GLUT family) [113]. It was shown that MM cell lines expressing high levels of GLUT1, and consequently increased glucose uptake, respond synergistically to the combinatorial treatment of bortezomib with the GLUT1 inhibitor STF-31 [112]. However, the mechanism of this synergistic effect was not investigated in depth.

**Figure 4:**
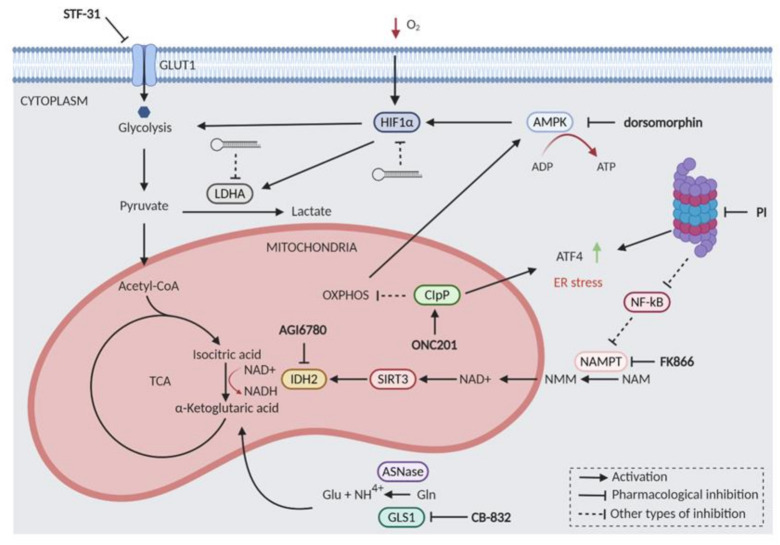
Proteasome inhibitors and metabolic pathways. Enhancement of glucose and glutamine metabolism is a hallmark of cancer cells, including multiple myeloma (MM). Targeting deranged cellular metabolism at various levels enhances the cytotoxicity of proteasome inhibitors in MM. Inhibition of the glucose transporter GLUT1 (STF-31) or the non-pharmacological inhibition of HIF1α and LDHA enzymes result in synergistic cell death with proteasome inhibitors (PI). The tricarboxylic acid (TCA) cycle enzyme IDH2 is synthetic lethal to PI through NAMPT/SIRT3/IDH2 pathway (AGI-6780, FK866). Degradation of glutamine can be targeted with Asparaginase (ASNase) or Glutaminase inhibitor (CB-832), affecting glutamate-dependent metabolites of TCA cycle. The cellular energy sensor AMPK is synthetically lethal to PI. OXPHOS inhibition by the mitochondrial protease ClpP (ONC201) leads to cellular stress and upregulation of transcriptional factor ATF4.

Isocitrate dehydrogenase 2 (IDH2) plays a key role in cellular metabolism and acts in the tricarboxylic acid (TCA) cycle catalyzing the reversible oxidative decarboxylation of isocitrate to α-ketoglutarate, NADPH, and CO2 [180]. Interestingly, it has been reported that IDH2 inhibition could increase the efficacy of conventional cancer therapies. Specifically, the genetic or pharmacologic inhibition of wild-type IDH2 synergized with PIs in MM and other hematological malignancies [42,117]. Mechanistically, PI treatment reduced the expression of nicotinamide phosphoribosyltransferase (NAMPT), thus limiting IDH2 activation through the NAD1-dependent deacetylase SIRT3. Consistently, the combination of carfilzomib with either NAMPT or SIRT3 inhibitors impaired IDH2 activity and increased MM cell death. Taken together, these findings indicate the NAMPT/SIRT3/IDH2 pathway as a major determinant of PIs responsiveness that can be exploited for new combination strategies aimed to enhance sensitivity and overcome resistance to PIs [42] (Figure 4).

A metabolomics profiling study showed that PI-resistant cells display massive alterations in cellular metabolism resulting in higher antioxidant capacity, higher redox homeostasis, and increased NAD+ levels [113]. This was a confirmation of previous studies where the NAD+ depleting agent FK866 was used with PIs to induce synergistic anti-MM cell death [42,117]. It was also demonstrated that a higher ATP/ADP ratio in ER allows higher protein folding capacity through more effective disulfide bond formation in resistant cells. The inhibition of protein disulfide isomerase has been shown to sensitize cells to PI in several studies [113,115]. Interestingly, in addition to increased TCA cycle and oxidative phosphorylation activities, PI-resistant cells also display altered mitochondria morphology [113,114]. As AMPK enzyme is inhibiting ATP-consuming pathways, its inhibitor dorsomorphin (also known as compound C) resulted highly synergistic with carfilzomib in samples from MM patients [147]. However, dorsomorphin has many other targets besides AMPK [181], hence the specificity of this particular synergy should be more deeply investigated. Noteworthy, activation of mitochondrial protease ClpP by imipridone ONC201 [182] leads to the loss of respiratory functions and triggers synergistic cytotoxicity with proteasome inhibitors in MM cell lines [183]. Moreover, a clinical trial is evaluating ONC201, ixazomib, and dexamethasone combination in RRMM patients (NCT03492138).

Additional studies suggested that glutamine is a critical fuel source that drives mitochondrial respiration in MM cells [114]. It has been previously shown that glutamine levels impact significantly on the metabolism of MM cells [176]. Glutamine is converted into glutamate and ammonia (NH4+) by GLS1 and GLS2 enzymes. Since it is recognized that MM cells are addicted to extracellular glutamine uptake because of poor glutamine synthetase (GS) expression [116], targeting the glutamine metabolism could open alternative therapeutic avenues. GLS catalyzes the conversion of glutamine to glutamate, which supports redox balance through glutathione biosynthesis, and serves as a major substrate for the mitochondrial tricarboxylic acid (TCA) cycle. Targeting glutamine metabolism with GLS1 specific inhibitor CB-839 synergistically enhanced the cytotoxic effects of PIs, with the most robust synergy being observed with carfilzomib [114]. Additionally, it was found that the glutaminolytic enzyme L-asparaginase, already used for the treatment of acute lymphoblastic leukemia (ALL), works synergistically with PIs in MM [116].

Although cancer cells usually prefer glycolysis and glutaminolysis over mitochondrial respiration [176], more recent studies are showing that drug-resistant cells and cancer stem cells depend heavily on oxidative phosphorylation (OXPHOS) for ATP generation [184]. Indeed, MM cells with the highest respiration rates could give rise to treatment-refractory disease [114]. Thus, targeting mitochondrial function in MM represents an attractive approach that will be more thoroughly investigated in the future (Figure 4). Sphingolipids are a class of lipids that exert pleiotropic cell signaling effects. Sphingolipid metabolism is often dysregulated in hematological malignancies and can confer resistance to many classes of drugs [185]. Ceramide is a central component of sphingolipid metabolism that is tightly regulated due to its pro-apoptotic effects. Sphingosine kinase 2 (SK2) inhibitors switch the chemical balance toward ceramide synthesis. In MM it has been described that the lipid metabolism of drug-resistant cells is swapped from lysolipids to sphingomyelins [113]. In line with this observation, SK2 inhibition synergizes with low dose bortezomib, most likely as a consequence of convergent ER stress and UPR activations [111]. This combination was also effective in vivo and had a good impact on bone disease. Proteasome inhibition by itself also has a great impact on metabolism which is manifested by the induction of amino acid biosynthesis, an antioxidant response, lipogenesis, and an increase in protein folding [177]. On the other side, as shown above, adaptive changes in MM cell metabolism may provide the basis of resistance to PI. Overall, metabolic pathways are attractive targets yet to be explored as effective therapeutic combinations with PIs (Figure 4).

### 3.7. Proteasome Inhibitors and Epigenetic Drugs

#### 3.7.1. Epigenetic Modifications as Druggable Targets in Multiple Myeloma

Epigenetics encompasses mechanisms that control heritable changes of gene expressions that do not entail modifications in DNA sequence. It involves remodeling of chromatin structure through covalent modifications of DNA, posttranslational histone alterations, and RNA interference [186,187]. DNA methylation occurs on the carbon-5 position of cytosine in a cytosine-phosphate-guanine (CpG) dinucleotide, resulting in 5-methylcytosine (5 mC) [188]. In addition, multiple reversible post-translational modifications (PTMs) are added on N-terminal tails of histones that are protruding from nucleosomes; among these, methylation, acetylation, phosphorylation, ubiquitination, sumoylation, and deamination at lysine, arginine, threonine, and serine residues. It is recognized that epigenetic changes cooperate with genetic alterations to drive the malignant phenotype of MM. Specifically, aberrant DNA methylation, histone modification profiles [189,190,191,192], and abnormal microRNA (miRNA) expressions [193,194,195] have been demonstrated to be instrumental to MM pathogenesis.

#### 3.7.2. Targeting DNA Methylation 

**Figure 5:**
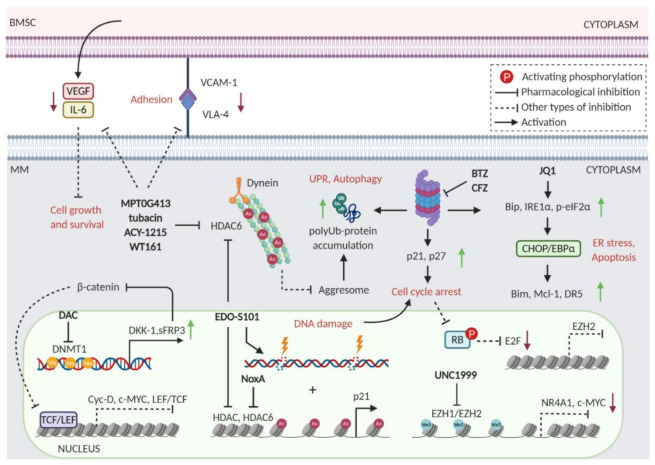
The molecular mechanisms underlying the effects of proteasome inhibitors (PIs) combination with epigenetic inhibitors. A plethora of anti-myeloma effects are observed by targeting epigenetic reprogrammers in combination with proteasome inhibition. HDAC6 specific inhibitors (MPT0G413, Tubacin, ACY-1215, WT161) lead to increased α-tubulin acetylation and to the inhibition of aggresomal pathways, thus activating unfolded protein response (UPR) and autophagy pathways. This mechanism is emphasized by proteasome inhibition. MPT0G413 can also affect MM cell growth, survival and adhesion to bone marrow stromal cells(BMSCs), through the inhibition of adhesion molecules and cytokines expression. The fusion molecule EDO-S101 acts as HDAC inhibitor as well as alkylating agent. EDO-S101 promotes polyUb-proteins accumulation, p21 expression, and induces DNA damage. Multiple pathways are responsible for the synergy between EDO-S101 and proteasome inhibitors, such as UPR hyper-activation, induction of autophagy, inhibition of cell cycle via upregulation of p21, and reduction of c-MYC expression. The BET inhibitor JQ1 in combination with carfilzomib enhances CHOP/EBPα-dependent Bim and Mcl-1 transcription, thus triggering ER stress and apoptosis. The DNA hypomethylating agent decitabine (DAC) demonstrated synergistic anti-MM effects in combinations with bortezomib. It is speculated that DAC inhibits β-catenin activity by promoting the expression of Wnt antagonists (DKK-1 and sFRP3). The EZH1/EZH2 inhibitor UNC1999 enhances the cytotoxicity of PI through a cooperative repression of MYC transcription.

DNA methylation is historically associated with transcriptional silencing. As in other malignancies, it has been observed that the methylation of promoters and CpG islands of tumor suppressor genes increases from MGUS to MM [196,197]. Moreover, hypermethylated sites in MM are also localized within B-cell-specific enhancer regions, thus suggesting that MM progression may occur in part, through dedifferentiation [198]. The level of DNA methylation is regulated by a set of enzymes called DNA methyltransferases 1, 3A, and 3B (DNMT1, DNMT3A, and DNMT3B) and the ten-eleven translocation (TET) protein. Inhibition of DNA methylation as a viable therapy for MM has been proved by several studies. DNA hypomethylating agents and pyrimidine nucleoside analogs such as 5-Azacytidine (AZA) and decitabine (DAC) demonstrated synergistic anti-MM effects in combinations with bortezomib [126,127,199,200]. The effectiveness of DAC/bortezomib combination was confirmed in MM xenograft models [201]. Specifically, the authors suggested the involvement of Wnt/β-catenin pathway, as a mediator of the synergic activity. The decreased methylation of Wnt pathway antagonists (DKK-1and sFRP-3) and DNMT3A promoters were proved responsible for the consequent reduction of nuclear β-catenin localization, and depletion of its downstream signaling [201] (Figure 5).

#### 3.7.3. Targeting Histone Methylation

A precise histones methylation balance, essential for oncogene and tumor suppressor genes regulation, is orchestrated by a plethora of enzymes such as histone methyltransferases (HMTs) and histone demethylases (HDMs). Consequently, aberrant histone methylation has been linked to tumorigenesis and MM progression [197]. Among the strategies to target histone methylation, the development of enhancer of zeste homolog 2 (EZH2) inhibitors has resulted in promising data, alone or in combination with proteasome inhibitors. To date, three EZH2 inhibitors GSK2816126, EPZ-6438, and CPI-1205 are undergoing phase I or II clinical trials in patients with MM and other types of tumors [202,203]. GSK2816126 (GSK-126) is a highly selective EZH2 inhibitor that blocks H3K27me3 and induces growth arrest and apoptosis in MM cells. Moreover, the authors demonstrated that GSK-126 eliminates cancer stem cells through the transcriptional upregulation of Wnt pathway antagonists. GSK-126 and bortezomib showed synergistic activity; however, the molecular mechanisms of these effects remain to be explained [204]. Interestingly, a novel small-molecule inhibitor of both EZH2 and EZH1, UNC1999, has recently shown a synergic activity in combination with bortezomib [125]. Importantly, UNC1999 enhanced the cytotoxicity of bortezomib and carfilzomib also in MM cell lines resistant to PI and dexamethasone. Moreover, this effect was confirmed in MM cells from patients and in xenograft models. Mechanistically, it was shown that UNC1999-bortezomib combination cooperatively represses MYC transcription, through the deregulation of NR4A1 and E2F1 factors, leading to induction of apoptosis and arrest of cell proliferation [125] (Figure 5).

#### 3.7.4. Targeting Histone Acetylation

Histone acetylation is one of the well-known PTM, involved in chromatin remodeling and gene regulation. Histones acetylation levels are efficiently balanced by histone acetyltransferase (HAT) and histone deacetylase (HDAC) enzymes. The latter are known to be implicated in several biological processes related to malignancies [205]. Furthermore, HDAC overexpression is observed in several types of human cancers [206]. Consistently, several HDAC inhibitors (HDACi) have been developed as antitumoral agents, including MM therapy [207]. The pan-HDAC inhibitors vorinostat, panobinostat and belinostat, and the class I-specific HDAC inhibitor romidepsin have been approved by the US Food and Drug Administration for the treatment of hematological cancers. All these drugs showed synergistic activity with bortezomib and were then proposed as effective agents to combine with proteasome inhibitors for the therapy of MM [122,208,209,210] (Table 2). Interestingly, a well-characterized mechanism of synergy between proteasome inhibitors and HDACi is through dual inhibition of the proteasome and aggresome pathways, with HDACi affecting non-epigenetic targets. However, other mechanisms have been described such as an increase in cytochrome-c release, caspase and poly-ADP-ribose polymerase cleavage, and inactivation of NF-κB, followed by apoptosis [208].

Since pan-HDACs display several side-effects, selective and potent HDACi have been developed, which are effective as single agents or in combination with other drugs. For example, Hideshima et al., showed that tubacin, a selective HDAC6 inhibitor, was able to enhance bortezomib cytotoxicity in MM cell lines and in patient-derived primary cells. They demonstrated that tubacin inhibits HDAC6 interaction with dynein, leading to accumulation of ubiquitinated proteins, and thus enhancing bortezomib-induced cytotoxicity, via JNK-caspase activation [121] (Figure 5).

Other HDAC6 inhibitors recently developed, such as ACY-1215, WT161, and MPT0G413 confirmed enhanced MM cytotoxicity in combination with PIs [119,121,211,212]. Besides the mechanism of action describe above, MPT0G413/bortezomib combination significantly down-regulated the expression of vascular cell adhesion molecule-1 (VCAM-1) and VLA-4, leading to a reduction of MM cell adhesion to BMSCs. Furthermore, MPT0G413/bortezomib combination decreased VEGF and IL-6 expression and secretion in the BM microenvironment, thus enforcing MM cell cycle arrest [119] (Figure 5).

Interestingly, hybrid compounds are novel promising drugs under evaluation to fight cancer cells. Indeed, EDO-S101 is a first-in-class fusion molecule derived from the alkylating agent bendamustine and the pan-HDAC inhibitor vorinostat [213]. A strong preclinical activity was shown in B-cell lymphoma and MM cell lines, MM primary samples, and mouse models of RRMM [214]. Importantly, EDO-S101 displayed potent synergic activity in combination with several anti-myeloma agents, including bortezomib [214]. It was further demonstrated that multiple pathways are responsible for the high synergy between EDO-S101 and bortezomib [118]. Among them, robust activation of the UPR, induction of autophagy, inhibition of cell cycle via upregulation of p21, and reduction of c-MYC expression were described (Figure 5).

Acetylated tails of histones are also recognized by epigenetic readers. Among these, the Bromodomain and Extra Terminal domain (BET) proteins include four members (BRDT, BRD2, BRD3, and BRD4) that mainly recognize acetylated lysine of histone 4. BET proteins act as scaffolds for other proteins to link promoters with enhancers and to facilitate gene transcription and elongation. BET proteins also recognize acetylated non-histone proteins, such as transcription factors, and can bind to other proteins in a bromodomain-independent manner [215]. Several preclinical studies imply a role of BET proteins in human cancers [215] and BET inhibitors have consistently emerged as promising cancer therapeutics [197]. The first two BET inhibitors described in 2010 were I-BET and JQ1, which act by interfering with the binding of bromodomain containing BET proteins to acetylated histones [216,217]. Interestingly, MYC transcriptional functions can be modulated pharmacologically by JQ1. Indeed, BRD4 is strongly enriched at immunoglobulin heavy-chain (IgH) enhancers in MM cells bearing IgH rearrangement at the MYC locus. JQ1 effectively depletes enhancer-bound BRD4 and promptly inhibits MYC transcription in a dose- and time-dependent manner with a consequent downregulation of the coordinated c-MYC transcriptional program, prompting cell-cycle arrest and cellular senescence [218]. However, earlier clinical trials showed limited activity of BET inhibitors as single-agent in patients with hematologic malignancies. Notably, the BET inhibitor JQ1 displayed synergistic activity in combination with carfilzomib in various cancer models, including MM [124]. The authors speculated that the JQ1/carfilzomib combination enhances ER stress leading to the activation of CHOP/EBPα, increased BIM transcription, and consequent apoptosis [124] (Figure 5). Accordingly, a recent study showed that BET inhibitors synergize with carfilzomib in multiple solid tumor cell lines [24]. In addition, Siegel et al. described that the selective BRD4 inhibitor CPI203 showed synergy with bortezomib in resistant cell lines as well as in a primary sample from RRMM patient. However, despite the combination induced antiproliferative effects and apoptosis, the molecular mechanisms of the synergy remain unknown [123].

In conclusion, several broad epigenetic reprogrammers have become pivotal for the treatment of MM, including pan-HDAC inhibitors and DNA hypomethylating agents. These drugs exert pleiotropic effects causing large-scale changes in gene expression and are effective in combination with proteasome inhibitors by acting directly on MM cells and the tumor microenvironment. However, broad reprogrammers are also more prone to side-effects and more difficult to schedule in combinatory therapies. More recently, the identification of specific genetic defects in epigenetic pathways in several type of cancers has led to the development of new targeted therapies. Among others, EZH2 inhibitors have shown promising beneficial outcomes in MM patients. Recent literature describes emerging epigenetic drugs with anti-tumoral effects, such as histone demethylases inhibitors [219]. Future investigation will point out whether a possible matching with PIs can enhance their efficacy.

### 3.8. Other Targets of Potential Therapeutic Interest

Recently, the nuclear exportin 1 (XPO1) inhibitor selinexor has been approved for the heavily pretreated MM patients who have received at least four prior lines of therapy [220]. Ongoing trials are investigating the efficacy of different PIs in combination with selinexor (Table 1). Observations from phase I/II studies indicate that selinexor/bortezomib combination did not affect ORR but increased progression-free survival (6.1 months vs. 3.7 months) of RRMM patients [221]. Selinexor, bortezomib, plus low-dose dexamethasone is currently in phase III trial in RRMM patients (NCT03110562). Selinexor, when used with bortezomib or carfilzomib, has the potential to overcome PI drug resistance in MM. Several molecular mechanisms of synergistic cell death induced by the combination of selinexor with PIs were suggested, such as the activation of Caspase-10 [222] or the accumulation of IκBα and thus the inactivation of the NF-κB pathway [223].

The frequent deregulation of the canonical Wnt/β-catenin pathway in MM by genetic and epigenetic means suggested that it could represent a suitable target for therapeutic intervention [224]. The Wnt/β-catenin pathway plays a key role in the interactions of MM cells with the bone marrow microenvironment. Specifically, MM cells secrete Wnt antagonists that contribute to the development of osteolytic lesions by impairing osteoblast differentiation. Increased β-catenin activation was consistently found in all MM cell lines. Consequently, exposure to the β-catenin inhibitor BC2059 triggered inhibition of proliferation and induction of apoptosis both in vitro and in vivo [128]. Most of the tested cell lines, as well as two patients’ samples, had shown synergistic death upon exposure with BC2059 and bortezomib. However, additional research is needed to understand the mechanism of this synergy.

## 4. Evolving Precision Medicine Using Combinatorial Drug Approach

Despite all progresses, MM is still an incurable disease, mostly due to its genetic heterogeneity. Unfortunately, many promising drugs have failed in patients, particularly when used as single agents. For example, even though the Ras/MAPK/ERK pathway is often deregulated in MM [3,5], trials with drug targeting the components of this pathway had a poor outcome. The main problem with single drug treatment is the activation of feedback loops within tumor cells, such as in the example of mTORC1 inhibitors [144]. Secondary, it can also be the result of crosstalk with other pathways. The well understood case is the cross-signaling of MAPK/ERK and PI3K/AKT pathways through activated Ras protein [225]. Next, it can often induce drug resistance. Finally, it can be the consequence of their interaction with the microenvironment [134]. Even though combinatorial treatments are nowadays the standard treatment for several malignancies, including MM, frequently they do not yield the expected results. Feedback loops pose main problem for a single drug treatment; however, targeting such loops, as in the example of MAPK/ERK and PI3K/AKT pathways, showed tolerability issues in clinical trials [225].

The traditional approaches of clinical trial design do not fit anymore in the personalized medicine era. Recently, integrated platforms have been designed to develop precision medicine approaches combining the identification of actionable genomic alterations with screening protocols. Moreover, the clonal heterogeneity of MM is a further challenge also for precision medicine, as therapy targeting only a subset of myeloma cells would not achieve optimal response [226]. Significant efforts have been made to map cancer-specific dependencies using genome-wide vulnerability screens and advanced computational algorithms for data analysis [227]. Such platforms will make integrated cancer data available to the community. Since there are many specific drugs already approved or in process of evaluation, the main obstacle is the development of disease-specific panels, which can not only detect the molecular alterations but also recognize their functional consequences. In line with this, master protocols with the scope to evaluate multiple treatments in several type of patients or disease within the same overall trial structure, are currently in development [228]. The ongoing trial MyDrug (NCT03732703) will enroll RRMM patients with specific mutations using genomics approaches. Drugs targeting the identified aberration will be used in combination with PI (ixazomib), IMiD (pomalidomide) and dexamethasone.

Great progress has been made in molecular profiling of MM cells; however, evaluation of patient immune system will also be of significant importance [226]. Combinatorial approaches using immunotherapy have become an important part of MM treatment. Various immunotherapeutic approaches such as antibody-drug conjugates, bispecific T-cell engagers (BiTEs), or CAR-T cells, mainly directed against BCMA and/or other plasma cell-specific antigens, are under clinical evaluation [229]. Therefore, immune profiling will have a great relevance for precision medicine in MM. In a recent pilot study using precision medicine in RRMM patients, several obstacles to the best capitalization of combinatorial drug research were identified [230]. Among these, insurance denials due to the high costs of therapies, the lack of standardized protocols for the detection of patient-specific aberrations, and the limited accuracy of predictions.

## 5. Conclusions 

In the present review, we have explored existing and emerging drug combinations with proteasome inhibitors in MM therapy. PIs are so far the most successful therapy for MM and due to their pleiotropic effects on multiple cellular pathways they represent an excellent partner for synergistic combinations. The development of high-throughput functional approaches, such as combinatorial screenings of drug, shRNA, siRNA, or CRISPR libraries, are revealing a multitude of new synthetic lethal interactions in MM. However, the comprehensive validation of these interactions is a bottleneck for their successful translation to clinics. As demonstrated, many promising preclinical results have failed in clinical trials. To diminish the probability of failure, more accurate ways of validation should be developed; possibly by using more accurate models, such as patient-derived tissues. Implementation of precision medicine into MM treatment would certainly lead to better outcomes by using most suitable therapeutic options. Nowadays, significant progress has been made by the simultaneous targeting of proteasomes and different aspects of MM-associated immune dysfunctions. Immunomodulatory drugs and monoclonal antibodies are so far the best synergistic partners to PIs based on the approval for the use in clinics. However, epigenetic drugs targeting either DNA methylation, histone modifiers/readers, or chromatin remodelers, exhibit pleiotropic anti-myeloma effects in combination with PIs. Importantly, they also act both on MM cells and their microenvironment. Finally, proteasome inhibition has a significant impact on MM metabolism. Therefore, the under-explored targeting of deranged metabolic hubs could represent a new avenue to identify effective therapeutic combinations with PIs.

## Figures and Tables

**Table 1:** Recent and ongoing clinical trials evaluating synergistic drug combinations with proteasome inhibitors (PI) in multiple myeloma (MM) therapy.

PI	Synergistic Partner	Signaling Pathway	Main Target	Phase/Stage	Reference
bortezomib (+dex)	lenalidomide	IMiDs	cereblon	III, ND	**NCT00644228** [63] *
ixazomib (+dex)				I/II, ND	NCT01217957 [65] *
				IV, RRMM	NCT03416374
				II, SMM	NCT02916771
				III, ND	NCT01850524
carfilzomib (+dex)				I, SMM	NCT01572480
	thalidomide			II, RRMM	NCT03140943
marizomib (+dex)	pomalidomide			I, RRMM	NCT02103335 *
carfilzomib (+dex)	elotuzumab	Antibody	CD319	II, RMM	NCT03155100
	daratumumab		CD38	III, RRMM	NCT03158688 [66]
bortezomib				III, RRMM	NCT02136134 [67]
carfilzomib (+dex)	isatuximab		CD38	III, RRMM	NCT03275285
bortezomib (+dex+CP)				II, MM	NCT04240054
carfilzomib	TTI-622	Other immunotherapy	CD47	I, RRMM	NCT03530683
	reovirus		JAM-A	I, RMM	NCT02101944
	reovirus+nivolumab		JAM-A, PD-L1	I, RRMM	NCT03605719
carfilzomib (+dex)	cabozantinib	Tyrosine kinase	c-MET	I/II, RRMM	NCT03201250
	ruxolitinib		JAK1/JAK2	I/II, RMM	NCT03773107
bortezomib	sorafenib		Raf, VEGFR, PDGFR	I, RMM	NCT00303797 [68] *
carfilzomib (+dex)	ibrutinib		BTK	I/II, RRMM	NCT01962792 [69] *
bortezomib (+dex)			BTK	II, RRMM	NCT02902965 *
	linisitinib (OSI-906)		IGF1R	I/II, RMM	NCT01672736 *
Bortezomib (+dex)	nelfinavir	PI3K/Akt/mTOR	Akt	II, MM	NCT02188537 [70] *
	perifosine		Akt	III, MM	NCT01002248 [71] *
	temsirolimus		mTORC1	I/II, RRMM	NCT00483262 [72] *
bortezomib (+dex)	MLN8237 (alisertib)	Cell cycle	Aurora A	I, RRMM	NCT01034553 [73] *
	PD0332991 (palbociclib)		Cdk4/6	I/II, RRMM	NCT00555906 [74] *
bortezomib	flavopiridol (alvociclib)		panCdk	I, RMM	NCT00082784 [75] *
	AT7519M		panCdk	I/II, RRMM	NCT01183949 [76] *
bortezomib (+dex)	dinaciclib		panCdk	I, RRMM	NCT01711528 *
carfilzomib (+dex)	TG02 citrate		panCdk	I, RRMM	NCT01204164 *
carfilzomib (+dex)	filanesib	Cytoskeletal signaling	KSP	I, RRMM	NCT01372540 [77] *
bortezomib (+dex)				I, RRMM	NCT01248923 [78] *
carfilzomib (+dex)	hydroxychloroquine	Stress	multiple	I, RRMM	NCT04163107
bortezomib				I, RRMM	NCT00568880 [79] *
bortezomib (+CP)	chloroquine			I, RRMM	NCT01438177 *
bortezomib (+dex)	ABT-888 (veliparib)		PARP	I, RRMM	NCT01495351 *
	ABT-199 (venetoclax)		BCL2	III, RRMM	NCT02755597 [80]
carfilzomib (+dex)				II, RRMM	NCT02899052 [81]
ixazomib (+dex)	pevonedistat		CRLs	I, RRMM	NCT03770260
bortezomib	KW-2478		HSP90	I, RRMM	NCT01063907 [82]
bortezomib (+dex)	ONC201	Metabolism	CLP	I/II, RRMM	NCT03492138
carfilzomib	panobinostat	Epigenetic	HDAC	I/II, RRMM	NCT01496118 [83]
bortezomib				III. RMM	NCT01023308 *
bortezomib (+dex)	ricolinostat		HDAC6	I/II, RRMM	NCT01323751 *
bortezomib	romidepsin		I-HDAC	II, RRMM	NCT00765102 *
bortezomib	selinexor	Other	XpoI	III, RRMM	NCT03110562
carfilzomib (+dex)				I, RRMM	NCT02199665 [84]
ixazomib (+dex)				I, RRMM, MM	NCT02831686

IMiDs—Immunomodulatory drugs, Dex—dexamethasone, CP—cyclophosphamide, -, ND—newly diagnosed MM, SMM—smoldering MM, RRMM—relapsed/refractory MM, * completed; designated signaling pathways are targeted by synergistic partners, **bold**—approved.

**Table 2:** Emerging drug combinations with proteasome inhibitors for the treatment of multiple myeloma.

PI	Synergistic Partner	Signaling Pathway *	Main Target	Phase/Stage	Reference
bortezomib	CC-292	Tyrosine kinase	BTK	cell lines, mouse MM model, primary samples	[96]
bortezomib	everolimus	PI3K/AKT/mTOR	FKBP12	cell lines, mouse MM model	[97]
	pp242		mTORC1 and 2	cell lines	[98]
carfilzomib	montelukast		mTOR pathway	cell lines, mouse MM model, primary samples	[99]
	copanlisib		PI3Kα, PI3Kδ	cell lines, primary samples	[100]
	TGR-1202		PI3Kδ	cell line	[101]
	TAS-117		AKT	cell lines, mouse MM model, primary samples	[102]
bortezomib	enzastaurin		PKC	cell lines, mouse MM model, primary samples	[103]
multiple	THZ1	Cell cycle	CDK7	cell lines, mouse MM model, primary samples	[104]
bortezomib	CASIN		Cdc42	cell lines, mouse MM model, primary samples	[105]
multiple	LU-102	Stress	β2-PI	cell lines	[106]
bortezomib	WRR139		NGLY	cell lines	[107]
	bafilomycin A1		Vacuolar ATPase	cell lines	[108]
	verapamil		calcium channel	cell lines	[109]
	metformin		GRP78	cell lines, mouse MM models, primary samples	[110]
bortezomib	K145	Metabolism	SK2	cell lines, mouse MM models	[111]
carfilzomib	AGI-6780		IDH2	cell lines, mouse MM models, primary samples	[42]
bortezomib	STF-31		GLUT1	cell lines	[112]
multiple	compound C		AMPK	cell lines	[113]
carfilzomib	CB-839		Glutaminase	cell lines	[114]
multiple	E61		PDI	cell lines, mouse MM model	[115]
bortezomib	L-asparaginase		Asn, Gln	cell lines, mouse MM models, primary samples	[116]
	FK866		NAD+	cell lines, mouse MM model	[117]
bortezomib	EDO-S101	Epigenetics	HDACs	cell lines, mouse MM models, primary samples	[118]
	MPT0G413		HDAC6	cell lines, mouse MM models	[119]
	nexturastat A		HDAC6	cell lines, mouse MM models	[120]
	tubacin		HDAC6	cell lines, primary samples	[121]
	WT161		HDAC6	cell lines, mouse MM models, primary sample	[121]
	belinostat		HDACs	cell lines, primary samples, osteloclast	[122]
	CPI203		BET	cell lines, primary samples	[123]
	JQ-1		BET	cell lines, mouse CRC models	[124]
	UNC1999		EZH1/EZH2	cell lines, mouse MM models, primary samples	[125]
	decitabine		DNMT	cell lines	[126]
	5-Azacytidine		DNMT	cell lines, primary samples	[127]
bortezomib	BC2059	Wnt/β catenin	β catenin	cell lines, primary samples	[128]

* Designated pathway is targeted by synergistic partner. PI- proteasome inihibitor

## Data Availability

Not applicable.

## Abbreviations

ALL—acute lymphoblastic leukemia
CP—cyclophosphamide
Dex—dexamethasone
ERAD—endoplasmic reticulum-associated protein degradation
IMiDs—immunomodulatory drugs
MGUS—monoclonal gammopathy of undetermined significance
MM—multiple myeloma
ND—newly diagnosed multiple myeloma
OXPHOS—oxidative phosphorylation
ORR—overall rate response
PI—proteasome inhibitors
RRMM—relapsed/refractory multiple myeloma
SMM—smoldering multiple myeloma
UPR—unfolded protein response
UPS—ubiquitin–proteasome system

## References

[1] Kumar S.K., Rajkumar V., Kyle R.A., Van Duin M., Sonneveld P., Mateos M.-V., Gay F., Anderson K.C. (2017). Multiple myeloma. Nat. Rev. Dis. Prim..

[2] Gandolfi S., Laubach J.P., Hideshima T., Chauhan D., Anderson K.C., Richardson P.G. (2017). The proteasome and proteasome inhibitors in multiple myeloma. Cancer Metastasis Rev..

[3] Manier S., Salem K.Z., Park J., Landau D.A., Getz G., Ghobrial I.M. (2017). Genomic complexity of multiple myeloma and its clinical implications. Nat. Rev. Clin. Oncol..

[4] Kumar S., Paiva B., Anderson K.C., Durie B., Landgren O., Moreau P., Munshi N., Lonial S., Bladé J., Mateos M.-V. (2016). International Myeloma Working Group consensus criteria for response and minimal residual disease assessment in multiple myeloma. Lancet Oncol..

[5] Maura F., Bolli N., Angelopoulos N., Dawson K.J., Leongamornlert D., Martincorena I., Mitchell T.J., Fullam A., Gonzalez S., Szalat R. (2019). Genomic landscape and chronological reconstruction of driver events in multiple myeloma. Nat. Commun..

[6] Mitchell J.S., Li N., Weinhold N., Försti A., Ali M., Van Duin M., Thorleifsson G., Johnson D.C., Chen B., Halvarsson B.-M. (2016). Genome-wide association study identifies multiple susceptibility loci for multiple myeloma. Nat. Commun..

[7] Gooding S., Olechnowicz S.W.Z., Morris E.V., Armitage A.E., Arezes J., Frost J., Repapi E., Edwards J.R., Ashley N., Waugh C. (2019). Transcriptomic profiling of the myeloma bone-lining niche reveals BMP signalling inhibition to improve bone disease. Nat. Commun..

[8] Rousseau A., Bertolotti A. (2018). Regulation of proteasome assembly and activity in health and disease. Nat. Rev. Mol. Cell Biol..

[9] Annunziata C.M., Davis R.E., Demchenko Y., Bellamy W., Gabrea A., Zhan F., Lenz G., Hanamura I., Wright G., Xiao W. (2007). Frequent Engagement of the Classical and Alternative NF-κB Pathways by Diverse Genetic Abnormalities in Multiple Myeloma. Cancer Cell.

[10] Nagel D., Vincendeau M., Eitelhuber A.C., Krappmann D. (2014). Mechanisms and consequences of constitutive NF-κB activation in B-cell lymphoid malignancies. Oncogene.

[11] Hussain A.R., Ahmed M., Ahmed S.O., Al-Thari S., Khan A.S., Razack S., Platanias L.C., Al-Kuraya K.S., Uddin S. (2009). Proteasome inhibitor MG-132 mediated expression of p27Kip1 via S-phase kinase protein 2 degradation induces cell cycle coupled apoptosis in primary effusion lymphoma cells. Leuk. Lymphoma.

[12] Qin J.-Z., Ziffra J., Stennett L., Bodner B., Bonish B.K., Chaturvedi V., Bennett F., Pollock P.M., Trent J.M., Hendrix M.J.C. (2005). Proteasome Inhibitors Trigger NOXA-Mediated Apoptosis in Melanoma and Myeloma Cells. Cancer Res..

[13] Obeng E.A., Carlson L.M., Gutman D.M., Harrington W.J., Lee K.P., Boise L.H. (2006). Proteasome inhibitors induce a terminal unfolded protein response in multiple myeloma cells. Blood.

[14] Zarfati M., Avivi I., Brenner B., Katz T., Aharon A. (2018). Extracellular vesicles of multiple myeloma cells utilize the proteasome inhibitor mechanism to moderate endothelial angiogenesis. Angiogenesis.

[15] Motegi A., Murakawa Y., Takeda S. (2009). The vital link between the ubiquitin–proteasome pathway and DNA repair: Impact on cancer therapy. Cancer Lett..

[16] Manasanch E.E., Orlowski R.Z. (2017). Proteasome inhibitors in cancer therapy. Nat. Rev. Clin. Oncol..

[17] Potts B.C., Albitar M.X., Anderson K.C., Baritaki S., Berkers C., Bonavida B., Chandra J., Chauhan D., Cusack J.C., Fenical W. (2011). Marizomib, a Proteasome Inhibitor for All Seasons: Preclinical Profile and a Framework for Clinical Trials. Curr. Cancer Drug Targets.

[18] Rajkumar S.V., Kumar S. (2020). Multiple myeloma current treatment algorithms. Blood Cancer J..

[19] Pinto V., Bergantim R., Caires H.R., Seca H., Guimarães J.E., Vasconcelos M.H. (2020). Multiple Myeloma: Available Therapies and Causes of Drug Resistance. Cancers.

[20] Anwer F., Gee K.M., Iftikhar A., Baig M., Russ A.D., Saeed S., Abu Zar M., Razzaq F., Carew J., Nawrocki S. (2019). Future of Personalized Therapy Targeting Aberrant Signaling Pathways in Multiple Myeloma. Clin. Lymphoma Myeloma Leuk..

[21] Ito S. (2020). Proteasome Inhibitors for the Treatment of Multiple Myeloma. Cancers.

[22] Roeten M.S.F., Cloos J., Jansen G. (2018). Positioning of proteasome inhibitors in therapy of solid malignancies. Cancer Chemother. Pharmacol..

[23] Raninga P.V., Lee A., Sinha D., Dong L.-F., Datta K.K., Lu X., Croft P.K.-D., Dutt M., Hill M., Pouliot N. (2020). Marizomib suppresses triple-negative breast cancer via proteasome and oxidative phosphorylation inhibition. Theranostics.

[24] Vangala J.R., Potluri A., Radhakrishnan S.K. (2020). BET Inhibitors Synergize with Carfilzomib to Induce Cell Death in Cancer Cells via Impairing Nrf1 Transcriptional Activity and Exacerbating the Unfolded Protein Response. Biomolecules.

[25] Turner J.G., Dawson J., Emmons M.F., Cubitt C.L., Kauffman M., Shacham S., Hazlehurst L.A., Sullivan D.M. (2013). CRM1 Inhibition Sensitizes Drug Resistant Human Myeloma Cells to Topoisomerase II and Proteasome Inhibitors both In Vitro and Ex Vivo. J. Cancer.

[26] Loewe S. (1953). The problem of synergism and antagonism of combined drugs. Arzneimittelforschung.

[27] Bliss C.I. (1939). The toxicity of poisons applied jointly. Ann. Appl. Biol..

[28] Gaddum J.H. (1948). Pharmacology.

[29] Chou T.-C., Talalay P. (1983). Analysis of combined drug effects: A new look at a very old problem. Trends Pharmacol. Sci..

[30] Schindler M. (2017). Theory of synergistic effects: Hill-type response surfaces as ‘null-interaction’ models for mixtures. Theor. Biol. Med Model..

[31] Twarog N.R., Stewart E., Hammill C.V., Shelat A.A. (2016). BRAID: A Unifying Paradigm for the Analysis of Combined Drug Action. Sci. Rep..

[32] Yadav B., Wennerberg K., Aittokallio T., Tang J. (2015). Searching for Drug Synergy in Complex Dose–Response Landscapes Using an Interaction Potency Model. Comput. Struct. Biotechnol. J..

[33] Zimmer A., Katzir I., Dekel E., Mayo A.E., Alon U. (2016). Prediction of multidimensional drug dose responses based on measurements of drug pairs. Proc. Natl. Acad. Sci. USA.

[34] Shi C.-X., Kortum K.M., Zhu Y.X., Jedlowski P., Braggio E., Bruins L.A., Votruba P.G., Luo M., Stewart R.A., Ahmann J.M. (2015). Crispr Sgrnas Genome-Wide Screen Identifies the Proteasome Regulatory Subunit PSMC6 As a Bortezomib Resistance Gene in Human Multiple Myeloma Cells. Blood.

[35] Sievers Q., Gasser J., Cowley G., Doench J.G., Fischer E., Ebert B.L. (2016). Genome-Scale Screen Reveals Genes Required for Lenalidomide-Mediated Degradation of Aiolos by CRL4-CRBN. Blood.

[36] Sudalagunta P., Silva M.C., Canevarolo R.R., Alugubelli R.R., DeAvila G., Tungesvik A., Perez L., Gatenby R., Gillies R., Baz R. (2020). A pharmacodynamic model of clinical synergy in multiple myeloma. EBioMedicine.

[37] Brioli A., Melchor L., Cavo M., Morgan G.J. (2014). The impact of intra-clonal heterogeneity on the treatment of multiple myeloma. Br. J. Haematol..

[38] Pemovska T., Bigenzahn J.W., Superti-Furga G. (2018). Recent advances in combinatorial drug screening and synergy scoring. Curr. Opin. Pharmacol..

[39] Zhu Y.X., Tiedemann R., Shi C.-X., Yin H., Schmidt J.E., Bruins L.A., Keats J.J., Braggio E., Sereduk C., Mousses S. (2011). RNAi screen of the druggable genome identifies modulators of proteasome inhibitor sensitivity in myeloma including CDK. Blood.

[40] Zhu Y.X., Yin H., Bruins L.A., Shi C.-X., Jedlowski P., Aziz M., Sereduk C., Kortuem K.M., Schmidt J.E., Champion M. (2015). RNA interference screening identifies lenalidomide sensitizers in multiple myeloma, including RSK2. Blood.

[41] Robert F., Roman W., Bramoullé A., Fellmann C., Roulston A., Shustik C., Porco J.A., Shore G.C., Sebag M., Pelletier J. (2014). Translation initiation factor eIF4F modifies the dexamethasone response in multiple myeloma. Proc. Natl. Acad. Sci. USA.

[42] Bergaggio E., Riganti C., Garaffo G., Vitale N., Mereu E., Bandini C., Pellegrino E., Pullano V., Omedè P., Todoerti K. (2019). IDH2 inhibition enhances proteasome inhibitor responsiveness in hematological malignancies. Blood.

[43] Tateno S., Iida M., Fujii S., Suwa T., Katayama M., Tokuyama H., Yamamoto J., Ito T., Sakamoto S., Handa H. (2020). Genome-wide screening reveals a role for subcellular localization of CRBN in the anti-myeloma activity of pomalidomide. Sci. Rep..

[44] Xie H., Gu Y., Wang W., Wang X., Ye X., Xin C., Lu M., Reddy B.A., Shu P. (2020). Silencing of SENP2 in Multiple Myeloma Induces Bortezomib Resistance by Activating NF-κB Through the Modulation of IκBα Sumoylation. Sci. Rep..

[45] Sievers Q.L., Gasser J.A., Cowley G.S., Fischer E.S., Ebert B.L. (2018). Genome-wide screen identifies cullin-RING ligase machinery required for lenalidomide-dependent CRL4CRBN activity. Blood.

[46] Shi C.-X., Kortüm K.M., Zhu Y.X., Bruins L.A., Jedlowski P., Votruba P.G., Luo M., Stewart R.A., Ahmann J., Braggio E. (2017). CRISPR Genome-Wide Screening Identifies Dependence on the Proteasome Subunit PSMC6 for Bortezomib Sensitivity in Multiple Myeloma. Mol. Cancer Ther..

[47] Liu J., Song T., Zhou W., Xing L., Wang S., Ho M., Peng Z., Tai Y.-T., Hideshima T., Anderson K.C. (2019). A genome-scale CRISPR-Cas9 screening in myeloma cells identifies regulators of immunomodulatory drug sensitivity. Leukemia.

[48] Babij C., Zhang Y., Kurzeja R.J., Munzli A., Shehabeldin A., Fernando M., Quon K., Kassner P.D., Ruefli-Brasse A.A., Watson V.J. (2011). STK33 Kinase Activity Is Nonessential in KRAS-Dependent Cancer Cells. Cancer Res..

[49] Lin A., Sheltzer J.M. (2020). Discovering and validating cancer genetic dependencies: Approaches and pitfalls. Nat. Rev. Genet..

[50] Pattanayak V., Lin S., Guilinger J.P., Ma E., Doudna J.A., Liu D.R. (2013). High-throughput profiling of off-target DNA cleavage reveals RNA-programmed Cas9 nuclease specificity. Nat. Biotechnol..

[51] Lin A., Giuliano C.J., Palladino A., John K.M., Abramowicz C., Yuan M.L., Sausville E.L., Lukow D.A., Liu L., Chait A.R. (2019). Off-target toxicity is a common mechanism of action of cancer drugs undergoing clinical trials. Sci. Transl. Med..

[52] Meyer C.T., Wooten D.J., Lopez C.F., Quaranta V. (2020). Charting the Fragmented Landscape of Drug Synergy. Trends Pharmacol. Sci..

[53] De Campos C.B., Meurice N., Petit J.L., Polito A.N., Zhu Y.X., Wang P., Bruins L.A., Wang X., Armenta I.D.L., Darvish S.A. (2020). “Direct to Drug” screening as a precision medicine tool in multiple myeloma. Blood Cancer J..

[54] Di Veroli G.Y., Fornari C., Wang D., Mollard S., Bramhall J.L., Richards F.M., Jodrell D.I. (2016). Combenefit: An interactive platform for the analysis and visualization of drug combinations. Bioinformatics.

[55] Preuer K., Lewis R.P.I., Hochreiter S., Bender A., Bulusu K.C., Klambauer G. (2018). DeepSynergy: Predicting anti-cancer drug synergy with Deep Learning. Bioinformatics.

[56] Zagidullin B., Aldahdooh J., Zheng S., Wang W., Wang Y., Saad J., Malyutina A., Jafari M., Tanoli Z., Pessia A. (2019). DrugComb: An integrative cancer drug combination data portal. Nucleic Acids Res..

[57] Ianevski A., He L., Aittokallio T., Tang J. (2017). SynergyFinder: A web application for analyzing drug combination dose–response matrix data. Bioinformatics.

[58] Ubels J., Sonneveld P., Van Vliet M.H., De Ridder J. (2020). Gene Networks Constructed Through Simulated Treatment Learning can Predict Proteasome Inhibitor Benefit in Multiple Myeloma. Clin. Cancer Res..

[59] Eichner R., Heider M., Fernández-Sáiz V., Van Bebber F., Garz A.-K., Lemeer S., Rudelius M., Targosz B.-S., Jacobs L., Knorn A.-M. (2016). Immunomodulatory drugs disrupt the cereblon–CD147–MCT1 axis to exert antitumor activity and teratogenicity. Nat. Med..

[60] Lu G., Middleton R.E., Sun H., Naniong M., Ott C.J., Mitsiades C.S., Wong K.-K., Bradner J.E., Kaelin W.G. (2014). The Myeloma Drug Lenalidomide Promotes the Cereblon-Dependent Destruction of Ikaros Proteins. Science.

[61] Ito T., Ando H., Suzuki T., Ogura T., Hotta K., Imamura Y., Yamaguchi Y., Handa H. (2010). Identification of a Primary Target of Thalidomide Teratogenicity. Science.

[62] Zhu Y.X., Braggio E., Shi C.-X., Bruins L.A., Schmidt J.E., Van Wier S., Chang X.-B., Bjorklund C.C., Fonseca R., Bergsagel P.L. (2011). Cereblon expression is required for the antimyeloma activity of lenalidomide and pomalidomide. Blood.

[63] Durie B.G.M., Hoering A., Abidi M.H., Rajkumar S.V., Epstein J., Kahanic S.P., Thakuri M., Reu F., Reynolds C.M., Sexton R. (2017). Bortezomib with lenalidomide and dexamethasone versus lenalidomide and dexamethasone alone in patients with newly diagnosed myeloma without intent for immediate autologous stem-cell transplant (SWOG S0777): A randomised, open-label, phase 3 trial. Lancet.

[64] Kumar S., Lee J.H., Lahuerta J.J., Morgan G., Richardson P.G., Crowley J., Haessler J., Feather J., Hoering A. (2011). Risk of progression and survival in multiple myeloma relapsing after therapy with IMiDs and bortezomib: A multicenter international myeloma working group study. Leukemia.

[65] Gupta N., Yang H., Hanley M.J., Zhang S., Liu R., Kumar S., Richardson P.G., Skacel T., Venkatakrishnan K. (2017). Dose and Schedule Selection of the Oral Proteasome Inhibitor Ixazomib in Relapsed/Refractory Multiple Myeloma: Clinical and Model-Based Analyses. Target. Oncol..

[66] Dimopoulos M., Quach H., Mateos M.-V., Landgren O., Leleu X., Siegel D., Weisel K., Yang H., Klippel Z., Zahlten-Kumeli A. (2020). Carfilzomib, dexamethasone, and daratumumab versus carfilzomib and dexamethasone for patients with relapsed or refractory multiple myeloma (CANDOR): Results from a randomised, multicentre, open-label, phase 3 study. Lancet.

[67] Chari A., Martinez-Lopez J., Mateos M.-V., Bladé J., Benboubker L., Oriol A., Arnulf B., Rodriguez-Otero P., Pineiro L., Jakubowiak A. (2019). Daratumumab plus carfilzomib and dexamethasone in patients with relapsed or refractory multiple myeloma. Blood.

[68] Kumar S.K., Jett J., Marks R., Richardson R., Quevedo F., Moynihan T., Croghan G., Markovic S.N., Bible K.C., Qin R. (2013). Phase 1 study of sorafenib in combination with bortezomib in patients with advanced malignancies. Investig. N. Drugs.

[69] Chari A., Larson S., Holkova B., Cornell R.F., Gasparetto C., Karanes C., Matous J.V., Niesvizky R., Valent J., Lunning M. (2018). Phase 1 trial of ibrutinib and carfilzomib combination therapy for relapsed or relapsed and refractory multiple myeloma. Leuk. Lymphoma.

[70] Driessen C., Müller R., Novak U., Cantoni N., Betticher D., Mach N., Rüfer A., Mey U., Samaras P., Ribi K. (2018). Promising activity of nelfinavir-bortezomib-dexamethasone in proteasome inhibitor–refractory multiple myeloma. Blood.

[71] Richardson P.G., Nagler A., Ben-Yehuda D., Badros A., Hari P.N., Hajek R., Spicka I., Kaya H., Leblanc R., Yoon S. (2020). Randomized, placebo-controlled, phase 3 study of perifosine combined with bortezomib and dexamethasone in patients with relapsed, refractory multiple myeloma previously treated with bortezomib. eJHaem.

[72] Ghobrial I.M., Weller E., Vij R., Munshi N.C., Banwait R., Bagshaw M., Schlossman R., LeDuc R., Chuma S., Kunsman J. (2011). Weekly bortezomib in combination with temsirolimus in relapsed or relapsed and refractory multiple myeloma: A multicentre, phase 1/2, open-label, dose-escalation study. Lancet Oncol..

[73] Rosenthal A., Kumar S., Hofmeister C., Laubach J., Vij R., Dueck A., Gano K., Stewart A.K. (2015). A Phase Ib Study of the combination of the Aurora Kinase Inhibitor Alisertib (MLN8237) and Bortezomib in Relapsed Multiple Myeloma. Br. J. Haematol..

[74] Niesvizky R., Badros A.Z., Costa L.J., Ely S.A., Singhal S.B., Stadtmauer E.A., Haideri N.A., Yacoub A., Hess G., Lentzsch S. (2015). Phase 1/2 study of cyclin-dependent kinase (CDK)4/6 inhibitor palbociclib (PD-0332991) with bortezomib and dexamethasone in relapsed/refractory multiple myeloma. Leuk. Lymphoma.

[75] Holkova B., Kmieciak M., Perkins E.B., Bose P., Baz R.C., Roodman G.D., Stuart R.K., Ramakrishnan V., Wan W., Peer C.J. (2014). Phase I Trial of Bortezomib (PS-341; NSC 681239) and “Nonhybrid” (Bolus) Infusion Schedule of Alvocidib (Flavopiridol; NSC 649890) in Patients with Recurrent or Refractory Indolent B-cell Neoplasms. Clin. Cancer Res..

[76] Raje N., Hari P.N., Landau H., Richardson P.G., Rosenblatt J., Couture R.N., Lyons J.F., Langford G., Yule M. (2013). A Phase I/II Open-Label Multicenter Study of the Cyclin Kinase Inhibitor AT7519M Alone and in Combination with Bortezomib in Patients with Previously Treated Multiple Myeloma. Blood.

[77] Lee H.C., Shah J.J., Feng L., Manasanch E.E., Lu R., Morphey A., Crumpton B., Patel K.K., Wang M.L., Alexanian R. (2019). A phase 1 study of filanesib, carfilzomib, and dexamethasone in patients with relapsed and/or refractory multiple myeloma. Blood Cancer J..

[78] Chari A., Htut M., Zonder J.A., Fay J.W., Jakubowiak A.J., Levy J.B., Lau K., Burt S.M., Tunquist B.J., Hilder B.W. (2016). A phase 1 dose-escalation study of filanesib plus bortezomib and dexamethasone in patients with recurrent/refractory multiple myeloma. Cancer.

[79] Vogl D.T., Stadtmauer E.A., Tan K.-S., Heitjan D.F., Davis L.E., Pontiggia L., Rangwala R., Piao S., Chang Y.C., Scott E.C. (2014). Combined autophagy and proteasome inhibition. Autophagy.

[80] Moreau P., Chanan-Khan A., Roberts A.W., Agarwal A.B., Facon T., Kumar S., Touzeau C., Punnoose E.A., Cordero J., Munasinghe W. (2017). Promising efficacy and acceptable safety of venetoclax plus bortezomib and dexamethasone in relapsed/refractory MM. Blood.

[81] Costa L.J., Stadtmauer E.A., Morgan G.J., Monohan G.P., Kovacsovics T., Burwick N., Jakubowiak A.J., Mobasher M., Freise K., Ross J.A. (2018). Phase 2 study of venetoclax plus carfilzomib and dexamethasone in patients with relapsed/refractory multiple myeloma. J. Clin. Oncol..

[82] Cavenagh J., Oakervee H., Baetiong-Caguioa P., Davies F., Gharibo M., Rabin N., Kurman M., Novak B., Shiraishi N., Nakashima D. (2017). A phase I/II study of KW-2478, an Hsp90 inhibitor, in combination with bortezomib in patients with relapsed/refractory multiple myeloma. Br. J. Cancer.

[83] Berdeja J.G., Hart L.L., Mace J.R., Arrowsmith E.R., Essell J.H., Owera R.S., Hainsworth J.D., Flinn I.W. (2015). Phase I/II study of the combination of panobinostat and carfilzomib in patients with relapsed/refractory multiple myeloma. Haematologica.

[84] Jakubowiak A.J., Jasielec J.K., Rosenbaum C.A., Cole C.E., Chari A., Mikhael J., Nam J., McIver A., Severson E., Stephens L.A. (2019). Phase 1 study of selinexor plus carfilzomib and dexamethasone for the treatment of relapsed/refractory multiple myeloma. Br. J. Haematol..

[85] Touzeau C., Moreau P., Dumontet C. (2017). Monoclonal antibody therapy in multiple myeloma. Leukemia.

[86] Soekojo C.Y., Ooi M., De Mel S., Chng W.J. (2020). Immunotherapy in Multiple Myeloma. Cells.

[87] San-Miguel J., Bladé J., Shpilberg O., Grosicki S., Maloisel F., Min C.-K., Zarzuela M.P., Robak T., Prasad S.V.S.S., Goh Y.T. (2014). Phase 2 randomized study of bortezomib-melphalan-prednisone with or without siltuximab (anti–IL-6) in multiple myeloma. Blood.

[88] Tai Y.-T., Dillon M., Song W., Leiba M., Li X.-F., Burger P., Lee A.I., Podar K., Hideshima T., Rice A.G. (2008). Anti-CS1 humanized monoclonal antibody HuLuc63 inhibits myeloma cell adhesion and induces antibody-dependent cellular cytotoxicity in the bone marrow milieu. Blood.

[89] Petrucci M.T., Vozella F. (2019). The Anti-CD38 Antibody Therapy in Multiple Myeloma. Cells.

[90] Spencer A.U., Lentzsch S., Weisel K., Avet-Loiseau H., Mark T.M., Spicka I., Masszi T., Lauri B., Levin M.-D., Bosi A. (2018). Daratumumab plus bortezomib and dexamethasone versus bortezomib and dexamethasone in relapsed or refractory multiple myeloma: Updated analysis of CASTOR. Haematologica.

[91] Krejcik J., Casneuf T., Nijhof I.S., Verbist B., Bald J., Plesner T., Syed K., Liu K., Van De Donk N.W.C.J., Weiss B.M. (2016). Daratumumab depletes CD38+ immune regulatory cells, promotes T-cell expansion, and skews T-cell repertoire in multiple myeloma. Blood.

[92] Van De Donk N.W., Usmani S.Z. (2018). CD38 Antibodies in Multiple Myeloma: Mechanisms of Action and Modes of Resistance. Front. Immunol..

[93] Attal M., Richardson P.G., Rajkumar S.V., San-Miguel J., Beksac M., Spicka I., Leleu X., Schjesvold F., Moreau P., Dimopoulos M.A. (2019). Isatuximab plus pomalidomide and low-dose dexamethasone versus pomalidomide and low-dose dexamethasone in patients with relapsed and refractory multiple myeloma (ICARIA-MM): A randomised, multicentre, open-label, phase 3 study. Lancet.

[94] Jelinek T., Paiva B., Hajek R. (2018). Update on PD-1/PD-L1 Inhibitors in Multiple Myeloma. Front. Immunol..

[95] Sun J., Muz B., Alhallak K., Markovic M., Gurley S., Wang Z., Guenthner N., Wasden K., Fiala M., King J. (2020). Targeting CD47 as a Novel Immunotherapy for Multiple Myeloma. Cancers.

[96] Eda H., Santo L., Cirstea D.D., Yee A.J., Scullen T.A., Nemani N., Mishima Y., Waterman P.R., Arastu-Kapur S., Evans E. (2014). A novel Bruton’s tyrosine kinase inhibitor CC-292 in combination with the proteasome inhibitor carfilzomib impacts the bone microenvironment in a multiple myeloma model with resultant antimyeloma activity. Leukemia.

[97] Li J., Liu Z., Li Y., Jing Q., Wang H., Liu H., Chen J., Feng J., Shao Q., Fu R. (2019). Everolimus shows synergistic antimyeloma effects with bortezomib via the AKT/mTOR pathway. J. Investig. Med..

[98] Hoang B., Frost P., Shi Y., Belanger E., Benavides A., Pezeshkpour G., Cappia S., Guglielmelli T., Gera J., Lichtenstein A. (2010). Targeting TORC2 in multiple myeloma with a new mTOR kinase inhibitor. Blood.

[99] Tong J., Yu Q., Xu W., Yu W., Wu C., Wu Y., Yan H. (2018). Montelukast enhances cytocidal effects of carfilzomib in multiple myeloma by inhibiting mTOR pathway. Cancer Biol. Ther..

[100] Okabe S., Tanaka Y., Tauchi T., Ohyashiki K. (2019). Copanlisib, a novel phosphoinositide 3-kinase inhibitor, combined with carfilzomib inhibits multiple myeloma cell proliferation. Ann. Hematol..

[101] Deng C., Lipstein M.R., Scotto L., Serrano X.O.J., Mangone M.A., Li S., Vendome J., Hao Y., Xu X., Deng S.-X. (2017). Silencing c-Myc translation as a therapeutic strategy through targeting PI3Kδ and CK1ε in hematological malignancies. Blood.

[102] Mimura N., Hideshima T., Shimomura T., Suzuki R., Ohguchi H., Rizq O., Kikuchi S., Yoshida Y., Cottini F., Jakubikova J. (2014). Selective and Potent Akt Inhibition Triggers Anti-Myeloma Activities and Enhances Fatal Endoplasmic Reticulum Stress Induced by Proteasome Inhibition. Cancer Res..

[103] Yang Y., Chen Y., Saha M.N., Chen J., Evans K., Qiu L., Reece D., Chen G.A., Chang H. (2014). Targeting phospho-MARCKS overcomes drug-resistance and induces antitumor activity in preclinical models of multiple myeloma. Leukemia.

[104] Zhang Y., Zhou L., Bandyopadhyay D., Sharma K., Allen A.J., Kmieciak M., Grant S. (2019). The Covalent CDK7 Inhibitor THZ1 Potently Induces Apoptosis in Multiple Myeloma Cells In Vitro and In Vivo. Clin. Cancer Res..

[105] Nguyen P., Chakrabarti J., Li Y., Kalim K.W., Zhang M., Zhang L., Zheng Y., Guo F. (2019). Rational Targeting of Cdc42 Overcomes Drug Resistance of Multiple Myeloma. Front. Oncol..

[106] Kraus M., Bader J., Geurink P.P., Weyburne E.S., Mirabella A.C., Silzle T., Shabaneh T.B., Van Der Linden W.A., De Bruin G., Haile S.R. (2015). The novel 2-selective proteasome inhibitor LU-102 synergizes with bortezomib and carfilzomib to overcome proteasome inhibitor resistance of myeloma cells. Haematologica.

[107] Tomlin F.M., Gerling-Driessen U.I.M., Liu Y.-C., Flynn R.A., Vangala J.R., Lentz C.S., Clauder-Muenster S., Jakob P., Mueller W.F., Ordoñez-Rueda D. (2017). Inhibition of NGLY1 Inactivates the Transcription Factor Nrf1 and Potentiates Proteasome Inhibitor Cytotoxicity. ACS Cent. Sci..

[108] Miyazawa K., Kawaguchi T., Moriya S., Ohtomo T., Che X.-F., Naito M., Itoh M., Tomoda A. (2011). Combined treatment with bortezomib plus bafilomycin A1 enhances the cytocidal effect and induces endoplasmic reticulum stress in U266 myeloma cells: Crosstalk among proteasome, autophagy-lysosome and ER stress. Int. J. Oncol..

[109] Meister S., Frey B., Lang V.R., Gaipl U.S., Schett G., Schlötzer-Schrehardt U., Voll R.E. (2010). Calcium Channel Blocker Verapamil Enhances Reticulum Stress and Death Induced by Proteasome Inhibition in Myeloma Cells. Neoplasia.

[110] Jagannathan S., Abdel-Malek M.A.Y., Malek E.E., Vad N.M., Latif T., Anderson K.C., Driscoll J.J. (2015). Pharmacologic screens reveal metformin that suppresses GRP78-dependent autophagy to enhance the anti-myeloma effect of bortezomib. Leukemia.

[111] Wallington-Beddoe C.T., Bennett M.K., VanDyke K., Davies L., Zebol J.R., Moretti P.A., Pitman M.R., Hewett D.R., Zannettino A.C., Pitson S.M. (2017). Sphingosine kinase 2 inhibition synergises with bortezomib to target myeloma by enhancing endoplasmic reticulum stress. Oncotarget.

[112] Matsumoto T., Jimi S., Migita K., Takamatsu Y., Hara S. (2016). Inhibition of glucose transporter 1 induces apoptosis and sensitizes multiple myeloma cells to conventional chemotherapeutic agents. Leuk. Res..

[113] Besse L., Besse A., Mendez-Lopez M., Vasickova K., Sedlackova M., Vanhara P., Kraus M., Bader J., Ferreira R.B., Castellano R.K. (2019). A metabolic switch in proteasome inhibitor-resistant multiple myeloma ensures higher mitochondrial metabolism, protein folding and sphingomyelin synthesis. Haematologica.

[114] Thompson R.M., Dytfeld D., Reyes L., Robinson R.M., Smith B., Manevich Y., Jakubowiak A., Komarnicki M., Przybylowicz-Chalecka A., Szczepaniak T. (2017). Glutaminase inhibitor CB-839 synergizes with carfilzomib in resistant multiple myeloma cells. Oncotarget.

[115] Robinson R.M., Reyes L., Duncan R.M., Bian H., Reitz A.B., Manevich Y., McClure J.J., Champion M.M., Chou C.J., Sharik M.E. (2018). Inhibitors of the protein disulfide isomerase family for the treatment of multiple myeloma. Leukemia.

[116] Bolzoni M., Chiu M., Accardi F., Vescovini R., Airoldi I., Storti P., Todoerti K., Agnelli L., Missale G., Andreoli R. (2016). Dependence on glutamine uptake and glutamine addiction characterize myeloma cells: A new attractive target. Blood.

[117] Cagnetta A., Cea M., Calimeri T., Acharya C., Fulciniti M., Tai Y.-T., Hideshima T., Chauhan D., Zhong M.Y., Patrone F. (2013). Intracellular NAD+ depletion enhances bortezomib-induced anti-myeloma activity. Blood.

[118] Besse L., Kraus M., Besse A., Bader J., Silzle T., Mehrling T., Driessen C. (2017). The first-in-class alkylating HDAC inhibitor EDO-S101 is highly synergistic with proteasome inhibition against multiple myeloma through activation of multiple pathways. Blood Cancer J..

[119] Huang F.-I., Wu Y.-W., Sung T.-Y., Liou J.-P., Lin M.-H., Pan S.-L., Yang C.-R. (2019). MPT0G413, A Novel HDAC6-Selective Inhibitor, and Bortezomib Synergistically Exert Anti-tumor Activity in Multiple Myeloma Cells. Front. Oncol..

[120] Sun X., Xie Y., Sun X., Yao Y., Li H., Li Z., Yao R., Xu K. (2019). The selective HDAC6 inhibitor Nexturastat A induces apoptosis, overcomes drug resistance and inhibits tumor growth in multiple myeloma. Biosci. Rep..

[121] Hideshima T., Qi J., Paranal R.M., Tang W., Greenberg E., West N., Colling M.E., Estiu G., Mazitschek R., Perry J.A. (2016). Discovery of selective small-molecule HDAC6 inhibitor for overcoming proteasome inhibitor resistance in multiple myeloma. Proc. Natl. Acad. Sci. USA.

[122] Feng R., Oton A., Mapara M.Y., Anderson G., Belani C., Lentzsch S. (2007). The histone deacetylase inhibitor, PXD101, potentiates bortezomib-induced anti-multiple myeloma effect by induction of oxidative stress and DNA damage. Br. J. Haematol..

[123] Siegel M.B., Liu S.Q., Davare M.A., Spurgeon S.E., Loriaux M.M., Druker B.J., Scott E.C., Tyner J.W. (2015). Small molecule inhibitor screen identifies synergistic activity of the bromodomain inhibitor CPI203 and bortezomib in drug resistant myeloma. Oncotarget.

[124] Qian G., Yao W., Zhang S., Bajpai R., Hall W.D., Shanmugam M., Lonial S., Sun S.-Y. (2018). Co-inhibition of BET and proteasome enhances ER stress and Bim-dependent apoptosis with augmented cancer therapeutic efficacy. Cancer Lett..

[125] Rizq O., Mimura N., Oshima M., Saraya A., Koide S., Kato Y., Aoyama K., Nakajima-Takagi Y., Wang C., Chiba T. (2017). Dual Inhibition of EZH2 and EZH1 Sensitizes PRC2-Dependent Tumors to Proteasome Inhibition. Clin. Cancer Res..

[126] Cao Y., Qiu G.-Q., Wu H.-Q., Wang Z.-L., Lin Y., Wu W., Xie X.-B., Gu W.-Y. (2016). Decitabine enhances bortezomib treatment in RPMI 8226 multiple myeloma cells. Mol. Med. Rep..

[127] Kiziltepe T., Hideshima T., Catley L., Raje N., Yasui H., Shiraishi N., Okawa Y., Ikeda H., Vallet S., Pozzi S. (2007). 5-Azacytidine, a DNA methyltransferase inhibitor, induces ATR-mediated DNA double-strand break responses, apoptosis, and synergistic cytotoxicity with doxorubicin and bortezomib against multiple myeloma cells. Mol. Cancer Ther..

[128] Savvidou I., Khong T., Cuddihy A., McLean C., Horrigan S., Spencer A. (2017). β-Catenin Inhibitor BC2059 Is Efficacious as Monotherapy or in Combination with Proteasome Inhibitor Bortezomib in Multiple Myeloma. Mol. Cancer Ther..

[129] Kelly K.R., Espitia C.M., Zhao W., Wendlandt E., Tricot G., Zhan F., Carew J.S., Nawrocki S.T. (2015). Junctional adhesion molecule-A is overexpressed in advanced multiple myeloma and determines response to oncolytic reovirus. Oncotarget.

[130] Solimando A.G., Da Vià M.C., Leone P., Borrelli P., Croci G.A., Tabares P., Brandl A., Di Lernia G., Bianchi F.P., Tafuri S. (2020). Halting the vicious cycle within the multiple myeloma ecosystem: Blocking JAM-A on bone marrow endothelial cells restores the angiogenic homeostasis and suppresses tumor progression. Haematologica.

[131] Sborov D.W., Nuovo G.J., Stiff A., Mace T., Lesinski G.B., Benson D.M., Efebera Y.A., Rosko A.E., Pichiorri F., Grever M.R. (2014). A Phase I Trial of Single-Agent Reolysin in Patients with Relapsed Multiple Myeloma. Clin. Cancer Res..

[132] Dona’ A., Viola D., Caserta E., Besi F., Sanchez J.F., Marcucci G., Keats J.J., Krishnan A.Y., Coffey M., Sborov D.W. (2019). Proteasome Inhibitors Impair the Innate Antiviral Immune Response and Potentiate Pelareorep-Based Viral Therapy in Multiple Myeloma. Blood.

[133] Hofmeister C.C., Sborov D.W., Viola D., Dona A., Nuovo G.J., Benson N.M., Lonial S., Kaufman J.L., Nooka A.K., Heffner L. (2018). Oncolytics Virus Replication Using Pelareorep (Reolysin) and Carfilzomib in Relapsed Myeloma Patients Increases PD-L1 Expression with Clinical Responses. Blood.

[134] Lind J., Czernilofsky F., Vallet S., Podar K. (2019). Emerging protein kinase inhibitors for the treatment of multiple myeloma. Expert Opin. Emerg. Drugs.

[135] Ramakrishnan V., Timm M., Haug J.L., Kimlinger T.K., Wellik L.E., Witzig T.E. (2010). Sorafenib, a Dual Raf Kinase/Vascular Endothelial Growth Factor Receptor Inhibitor Has Significant Anti-Myeloma Activity and Synergizes with Common Anti-Myeloma Drugs. Oncogene.

[136] Suominen M.I., Fagerlund K.M., Alhoniemi E., Rissanen J.P., Halleen J.M., Aftab D.T. (2015). Abstract 786: Effects of combination treatment with cabozantinib and bortezomib in the 5TGM1 murine multiple myeloma model. Cancer Res..

[137] Kuhn D.J., Berkova Z., Jones R.J., Woessner R., Bjorklund C.C., Ma W., Davis R.E., Lin P., Wang H., Madden T.L. (2012). Targeting the insulin-like growth factor-1 receptor to overcome bortezomib resistance in preclinical models of multiple myeloma. Blood.

[138] Singh S.P., Dammeijer F., Hendriks R.W. (2018). Role of Bruton’s tyrosine kinase in B cells and malignancies. Mol. Cancer.

[139] Tai Y.-T., Chang B.Y., Kong S.-Y., Fulciniti M., Yang G., Calle Y., Hu Y., Lin J., Zhao J.-J., Cagnetta A. (2012). Bruton tyrosine kinase inhibition is a novel therapeutic strategy targeting tumor in the bone marrow microenvironment in multiple myeloma. Blood.

[140] Yu L., Mohamed A.J., Simonson O.E., Vargas L., Blomberg K.E.M., Björkstrand B., Arteaga H.J., Nore B.F., Smith C.I.E. (2008). Proteasome-dependent autoregulation of Bruton tyrosine kinase (Btk) promoter via NF-κB. Blood.

[141] Murray M.Y., Zaitseva L., Auger M.J., Craig J.I., MacEwan D.J., A Rushworth S., Bowles K.M. (2015). Ibrutinib inhibits BTK-driven NF-κB p65 activity to overcome bortezomib-resistance in multiple myeloma. Cell Cycle.

[142] Richardson P.G., Bensinger W.I., Huff C.A., Costello C.L., Lendvai N., Berdeja J.G., Anderson L.D., Siegel D.S., Lebovic D., Jagannath S. (2018). Ibrutinib alone or with dexamethasone for relapsed or relapsed and refractory multiple myeloma: Phase 2 trial results. Br. J. Haematol..

[143] Hu J., Hu W.-X. (2018). Targeting signaling pathways in multiple myeloma: Pathogenesis and implication for treatments. Cancer Lett..

[144] Ra S., Dm S. (2017). MTOR Signaling in Growth, Metabolism and Disease. Cell.

[145] Thorpe L.M., Yuzugullu H., Zhao J.J. (2015). PI3K in cancer: Divergent roles of isoforms, modes of activation and therapeutic targeting. Nat. Rev. Cancer.

[146] Nass J., Efferth T. (2018). Drug targets and resistance mechanisms in multiple myeloma. Cancer Drug Resist..

[147] Kim J., Guan K.-L. (2019). mTOR as a central hub of nutrient signalling and cell growth. Nat. Cell Biol..

[148] Hideshima T., Mitsiades C.S., Tonon G., Richardson P.G., Anderson K.C. (2007). Understanding multiple myeloma pathogenesis in the bone marrow to identify new therapeutic targets. Nat. Rev. Cancer.

[149] Shin S., Wolgamott L., Roux P.P., Yoon S.-O. (2014). Casein Kinase 1ε Promotes Cell Proliferation by Regulating mRNA Translation. Cancer Res..

[150] Richardson P.G., Eng C., Kolesar J., Hideshima T., Anderson K.C. (2012). Perifosine, an oral, anti-cancer agent and inhibitor of the Akt pathway: Mechanistic actions, pharmacodynamics, pharmacokinetics, and clinical activity. Expert Opin. Drug Metab. Toxicol..

[151] Richardson P.G., Wolf J., Jakubowiak A., Zonder J., Lonial S., Irwin D., Densmore J., Krishnan A., Raje N., Bar M. (2011). Perifosine Plus Bortezomib and Dexamethasone in Patients with Relapsed/Refractory Multiple Myeloma Previously Treated with Bortezomib: Results of a Multicenter Phase I/II Trial. J. Clin. Oncol..

[152] Krauß M., Bader J., Overkleeft H., Driessen C. (2013). Nelfinavir augments proteasome inhibition by bortezomib in myeloma cells and overcomes bortezomib and carfilzomib resistance. Blood Cancer J..

[153] Podar K., Raab M.S., Zhang J., McMillin D., Breitkreutz I., Tai Y.-T., Lin B.K., Munshi N., Hideshima T., Chauhan D. (2006). Targeting PKC in multiple myeloma: In Vitro and In Vivo effects of the novel, orally available small-molecule inhibitor enzastaurin (LY317615.HCl). Blood.

[154] Maes A., Menu E., De Veirman K., Maes K., Erkerken K.V., De Bruyne E. (2017). The therapeutic potential of cell cycle targeting in multiple myeloma. Oncotarget.

[155] Dikic I. (2017). Proteasomal and Autophagic Degradation Systems. Annu. Rev. Biochem..

[156] Pines J. (2011). Cubism and the cell cycle: The many faces of the APC/C. Nat. Rev. Mol. Cell Biol..

[157] Kwiatkowski N.P., Zhang T., Rahl P.B., Abraham B.J., Reddy J., Ficarro S.B., Dastur A., Amzallag A., Ramaswamy S., Tesar B. (2014). Targeting transcription regulation in cancer with a covalent CDK7 inhibitor. Nat. Cell Biol..

[158] Stengel K., Zheng Y. (2011). Cdc42 in oncogenic transformation, invasion, and tumorigenesis. Cell. Signal..

[159] Nikesitch N., Lee J.M., Ling S., Roberts T.L. (2018). Endoplasmic reticulum stress in the development of multiple myeloma and drug resistance. Clin. Transl. Immunol..

[160] Zhang L., Fok J.H., Davies F.E. (2014). Heat shock proteins in multiple myeloma. Oncotarget.

[161] Ishii T., Seike T., Nakashima T., Juliger S., Maharaj L., Soga S., Akinaga S., Cavenagh J., Joel S.P., Shiotsu Y. (2012). Anti-tumor activity against multiple myeloma by combination of KW-2478, an Hsp90 inhibitor, with bortezomib. Blood Cancer J..

[162] Eugênio A.I.P., Fook-Alves V.L., De Oliveira M.B., Fernando R.C., Zanatta D.B., Strauss B.E., Silva M.R.R., Porcionatto M.A., Colleoni G.W.B. (2017). Proteasome and heat shock protein 70 (HSP70) inhibitors as therapeutic alternative in multiple myeloma. Oncotarget.

[163] Jarauta V., Jaime P., Gonzalo O., de Miguel D., Ramírez-Labrada A., Martínez-Lostao L., Anel A., Pardo J., Marzo I., Naval J. (2016). Inhibition of autophagy with chloroquine potentiates carfilzomib-induced apoptosis in myeloma cells In Vitro and In Vivo. Cancer Lett..

[164] Baranowska K., Misund K., Starheim K.K., Holien T., Johansson I., Darvekar S., Buene G., Waage A., Bjørkøy G., Sundan A. (2016). Hydroxychloroquine potentiates carfilzomib toxicity towards myeloma cells. Oncotarget.

[165] Lu Y., Wang Y., Xu H., Shi C., Jin F., Li W. (2018). Profilin 1 induces drug resistance through Beclin1 complex-mediated autophagy in multiple myeloma. Cancer Sci..

[166] Pihán P., Carreras-Sureda A., Hetz C. (2017). BCL-2 family: Integrating stress responses at the ER to control cell demise. Cell Death Differ..

[167] Hoang B., Benavides A., Shi Y., Frost P., Lichtenstein A. (2009). Effect of autophagy on multiple myeloma cell viability. Mol. Cancer Ther..

[168] Punnoose E.A., Leverson J.D., Peale F., Boghaert E.R., Belmont L.D., Tan N., Young A., Mitten M., Ingalla E., Darbonne W.C. (2016). Expression Profile of BCL-2, BCL-XL, and MCL-1 Predicts Pharmacological Response to the BCL-2 Selective Antagonist Venetoclax in Multiple Myeloma Models. Mol. Cancer Ther..

[169] Harrison M.S., Cavo M., De La Rubia J., Popat R., Gasparetto C., Hungria V.T., Salwender H., Suzuki K., Kim I., Moreau P. (2019). T(11;14) and High BCL2 Expression Are Predictive Biomarkers of Response to Venetoclax in Combination with Bortezomib and Dexamethasone in Patients with Relapsed/Refractory Multiple Myeloma: Biomarker Analyses from the Phase 3 Bellini Study. Blood.

[170] Boccon-Gibod C., Talbot A., Le Bras F., Frenzel L., Royer B., Harel S., Lombion N., Belhadj K., Cuccuini W., Arnulf B. (2020). Carfilzomib, venetoclax and dexamethasone for relapsed/refractory multiple myeloma. Br. J. Haematol..

[171] Neri P., Ren L., Gratton K., Stebner E., Johnson J., Klimowicz A., Duggan P., Tassone P., Mansoor A., Stewart U.A. (2011). Bortezomib-induced “BRCAness” sensitizes multiple myeloma cells to PARP inhibitors. Blood.

[172] Yoshida T., Ri M., Kanamori T., Aoki S., Ashour R., Kinoshita S., Narita T., Totani H., Masaki A., Ito A. (2018). Potent anti-tumor activity of a syringolin analog in multiple myeloma: A dual inhibitor of proteasome activity targeting β2 and β5 subunits. Oncotarget.

[173] Soucy T.A., Smith P.G., Milhollen M.A., Berger A.J., Gavin J.M., Adhikari S., Brownell J.E., Burke K.E., Cardin D.P., Critchley S. (2009). An inhibitor of NEDD8-activating enzyme as a new approach to treat cancer. Nat. Cell Biol..

[174] Gu Y., Kaufman J.L., Bernal L., Torre C., Matulis S.M., Harvey R.D., Chen J., Sun S.-Y., Boise L.H., Lonial S. (2014). MLN4924, an NAE inhibitor, suppresses AKT and mTOR signaling via upregulation of REDD1 in human myeloma cells. Blood.

[175] Muraoka H., Yoshimura C., Kawabata R., Tsuji S., Hashimoto A., Ochiiwa H., Nakagawa F., Fujioka Y., Matsuo K., Ohkubo S. (2019). Activity of TAS4464, a novel NEDD8 activating enzyme E1 inhibitor, against multiple myeloma via inactivation of nuclear factor κB pathways. Cancer Sci..

[176] El Arfani C., De Veirman K., Maes K., De Bruyne E., Menu E. (2018). Metabolic Features of Multiple Myeloma. Int. J. Mol. Sci..

[177] Maiso P., Huynh D., Moschetta M., Sacco A., Aljawai Y., Mishima Y., Asara J.M., Roccaro A.M., Kimmelman A.C., Ghobrial I.M. (2015). Metabolic Signature Identifies Novel Targets for Drug Resistance in Multiple Myeloma. Cancer Res..

[178] Denko N.C. (2008). Hypoxia, HIF and Metabolism in the Solid Tumour. Nat. Rev. Cancer.

[179] El Hout M., Cosialls E., Mehrpour M., Hamaï A. (2020). Crosstalk between autophagy and metabolic regulation of cancer stem cells. Mol. Cancer.

[180] Bergaggio E., Piva R. (2019). Wild-Type IDH Enzymes as Actionable Targets for Cancer Therapy. Cancers.

[181] Dasgupta B., Seibel W., Neumann D., Viollet B. (2018). Compound C/Dorsomorphin: Its Use and Misuse as an AMPK Inhibitor. AMPK: Methods and Protocols.

[182] Wang S., Dougan D.A. (2019). The Direct Molecular Target for Imipridone ONC201 Is Finally Established. Cancer Cell.

[183] Tu Y.-S., Yong-Sheng T., Liu H., Lee H.C., Wang H., Ishizawa J., Allen J.E., Andreeff M., Orlowski R.Z., Davis R.E. (2017). The Imipridone ONC201 Induces Apoptosis and Overcomes Chemotherapy Resistance by Up-Regulation of Bim in Multiple Myeloma. Neoplasia.

[184] Ma L., Zong X. (2020). Metabolic Symbiosis in Chemoresistance: Refocusing the Role of Aerobic Glycolysis. Front. Oncol..

[185] Lewis A.C., Wallington-Beddoe C.T., Powell J.A., Pitson S.M. (2018). Targeting sphingolipid metabolism as an approach for combination therapies in haematological malignancies. Cell Death Discov..

[186] Helin K., Dhanak D. (2013). Chromatin proteins and modifications as drug targets. Nat. Cell Biol..

[187] Lippman Z., Martienssen R. (2004). The role of RNA interference in heterochromatic silencing. Nat. Cell Biol..

[188] Smith Z.D., Meissner A. (2013). DNA methylation: Roles in mammalian development. Nat. Rev. Genet..

[189] Chim C.S. (2010). Updated survivals and prognostic factor analysis in myeloma treated by a staged approach use of bortezomib/thalidomide/dexamethasone in transplant eligible patients. J. Transl. Med..

[190] Walker B.A., Wardell C.P., Chiecchio L., Smith E.M., Boyd K.D., Neri A., Davies F.E., Ross F.M., Morgan G.J. (2011). Aberrant global methylation patterns affect the molecular pathogenesis and prognosis of multiple myeloma. Blood.

[191] Sive J.I., Feber A., Smith D., Quinn J., Beck S., Yong K. (2015). Global hypomethylation in myeloma is associated with poor prognosis. Br. J. Haematol..

[192] Martinez-Garcia E., Popovic R., Min D.-J., Sweet S.M.M., Thomas P.M., Zamdborg L., Heffner A., Will C., Lamy L., Staudt L.M. (2011). The MMSET histone methyl transferase switches global histone methylation and alters gene expression in t(4;14) multiple myeloma cells. Blood.

[193] Pichiorri F., Suh S.-S., Ladetto M., Kuehl M., Palumbo T., Drandi D., Taccioli C., Zanesi N., Alder H., Hagan J.P. (2008). MicroRNAs regulate critical genes associated with multiple myeloma pathogenesis. Proc. Natl. Acad. Sci. USA.

[194] Attia H.R., Abdelrahman A.H., Ibrahim M.H., Eid M.M., Eid O.M., Sallam M.T., El Gammal M.M., Kamel M.M. (2020). Altered Expression of MicroRNAs in the Bone Marrow of Multiple Myeloma Patients and their Relationship to Cytogenetic Aberrations. Curr. Pharm. Biotechnol..

[195] Handa H., Murakami Y., Ishihara R., Kimura-Masuda K., Masuda Y. (2019). The Role and Function of microRNA in the Pathogenesis of Multiple Myeloma. Cancers.

[196] Geraldes C., Gonçalves A.C., Cortesão E., Pereira M.I., Roque A., Paiva A., Ribeiro L., Nascimento-Costa J.M., Sarmento-Ribeiro A.B. (2016). Aberrant p15, p16, p53, and DAPK Gene Methylation in Myelomagenesis: Clinical and Prognostic Implications. Clin. Lymphoma Myeloma Leuk..

[197] Alzrigat M., Párraga A.A., Jernberg-Wiklund H. (2018). Epigenetics in multiple myeloma: From mechanisms to therapy. Semin. Cancer Biol..

[198] Agirre X., Castellano G., Pascual M., Heath S., Kulis M., Segura V., Bergmann A., Esteve A., Merkel A., Raineri E. (2015). Whole-epigenome analysis in multiple myeloma reveals DNA hypermethylation of B cell-specific enhancers. Genome Res..

[199] Turcan S., Fabius A.W.M., Borodovsky A., Pedraza A., Brennan C., Huse J., Viale A., Riggins G.J., Chan T.A. (2013). Efficient induction of differentiation and growth inhibition in IDH1 mutant glioma cells by the DNMT Inhibitor Decitabine. Oncotarget.

[200] Ding L., Qiu L., Zhang J., Guo B. (2009). Camptothecin-induced cell proliferation inhibition and apoptosis enhanced by DNA methyltransferase inhibitor, 5-aza-2’-deoxycytidine. Biol. Pharm. Bull..

[201] Jin Y., Xu L., Wu X., Feng J., Shu M., Gu H., Gao G., Zhang J., Dong B., Chen X. (2019). Synergistic Efficacy of the Demethylation Agent Decitabine in Combination with the Protease Inhibitor Bortezomib for Treating Multiple Myeloma through the Wnt/β-Catenin Pathway. Oncol. Res. Featur. Preclin. Clin. Cancer Ther..

[202] Yap T.A., Winter J.N., Giulino-Roth L., Longley J., Lopez J., Michot J.-M., Leonard J.P., Ribrag V., McCabe M.T., Creasy C.L. (2019). Phase I Study of the Novel Enhancer of Zeste Homolog 2 (EZH2) Inhibitor GSK2816126 in Patients with Advanced Hematologic and Solid Tumors. Clin. Cancer Res..

[203] Yap T.A., Johnson P.W.M., Winter J., Leonard J., Giulino-Roth L., Horner T., Radswillas K., Carver J., Dhar A. (2016). A phase I, open-label study of GSK2816126, an enhancer of zeste homolog 2 (EZH2) inhibitor, in patients with relapsed/refractory diffuse large B-cell lymphoma (DLBCL), transformed follicular lymphoma (tFL), other non-Hodgkin’s lymphomas (NHL), multiple myeloma (MM) and solid tumor. J. Clin. Oncol..

[204] Zeng D., Liu M., Pan J. (2017). Blocking EZH2 methylation transferase activity by GSK126 decreases stem cell-like myeloma cells. Oncotarget.

[205] Imai Y., Hirano M., Kobayashi M., Futami M., Tojo A. (2019). HDAC Inhibitors Exert Anti-Myeloma Effects through Multiple Modes of Action. Cancers.

[206] Witt O., Deubzer H.E., Milde T., Oehme I. (2009). HDAC family: What are the cancer relevant targets?. Cancer Lett..

[207] Seval G.C., Beksac M. (2019). A comparative safety review of histone deacetylase inhibitors for the treatment of myeloma. Expert Opin. Drug Saf..

[208] Pei X.-Y., Dai Y., Grant S. (2004). Synergistic Induction of Oxidative Injury and Apoptosis in Human Multiple Myeloma Cells by the Proteasome Inhibitor Bortezomib and Histone Deacetylase Inhibitors. Clin. Cancer Res..

[209] Kikuchi J., Wada T., Shimizu R., Izumi T., Akutsu M., Mitsunaga K., Noborio-Hatano K., Nobuyoshi M., Ozawa K., Kano Y. (2010). Histone deacetylases are critical targets of bortezomib-induced cytotoxicity in multiple myeloma. Blood.

[210] Catley L., Weisberg E., Kiziltepe T., Tai Y.-T., Hideshima T., Neri P., Tassone P., Atadja P., Chauhan D., Munshi N.C. (2006). Aggresome induction by proteasome inhibitor bortezomib and α-tubulin hyperacetylation by tubulin deacetylase (TDAC) inhibitor LBH589 are synergistic in myeloma cells. Blood.

[211] Santo L., Hideshima T., Kung A.L., Tseng J.-C., Tamang D., Yang M., Jarpe M., Van Duzer J.H., Mazitschek R., Ogier W.C. (2012). Preclinical activity, pharmacodynamic, and pharmacokinetic properties of a selective HDAC6 inhibitor, ACY-1215, in combination with bortezomib in multiple myeloma. Blood.

[212] Menden M.P., Wang D., Mason M.J., Szalai B., Bulusu K.C., Guan Y., Yu T., Kang J., Jeon M. (2019). Community assessment to advance computational prediction of cancer drug combinations in a pharmacogenomic screen. Nat. Commun..

[213] Mehrling T., Chen Y. (2015). The Alkylating-HDAC Inhibition Fusion Principle: Taking Chemotherapy to the Next Level with the First in Class Molecule EDO-S101. Anti-Cancer Agents Med. Chem..

[214] López-Iglesias A.-A., Herrero A.B., Chesi M., San-Segundo L., González-Méndez L., Hernández-García S., Misiewicz-Krzeminska I., Quwaider D., Martín-Sánchez M., Primo D. (2017). Preclinical anti-myeloma activity of EDO-S101, a new bendamustine-derived molecule with added HDACi activity, through potent DNA damage induction and impairment of DNA repair. J. Hematol. Oncol..

[215] Stathis A., Bertoni F. (2017). BET Proteins as Targets for Anticancer Treatment. Cancer Discov..

[216] Nicodeme E., Jeffrey K.L., Schaefer U., Beinke S., Dewell S., Chung C.-W., Chandwani R., Marazzi I., Wilson P., Coste H. (2010). Suppression of inflammation by a synthetic histone mimic. Nat. Cell Biol..

[217] Filippakopoulos P., Qi J., Picaud S., Shen Y., Smith M.C., Fedorov O., Morse E.M., Keates T., Hickman T.T., Felletar I. (2010). Selective inhibition of BET bromodomains. Nature.

[218] Delmore J.E., Issa G.C., Lemieux M.E., Rahl P.B., Shi J., Jacobs H.M., Kastritis E., Gilpatrick T., Paranal R.M., Qi J. (2011). BET Bromodomain Inhibition as a Therapeutic Strategy to Target c-Myc. Cell.

[219] De Smedt E., Lui H., Maes K., De Veirman K., Menu E., Vanderkerken K., De Bruyne E. (2018). The Epigenome in Multiple Myeloma: Impact on Tumor Cell Plasticity and Drug Response. Front. Oncol..

[220] U.S. Food & Drug Administration https://www.fda.gov/drugs/resources-information-approved-drugs/fda-grants-accelerated-approval-selinexor-multiple-myeloma.

[221] Bahlis N.J., Sutherland H., White D., Sebag M., Lentzsch S., Kotb R., Venner C.P., Gasparetto C., Del Col A., Neri P. (2018). Selinexor plus low-dose bortezomib and dexamethasone for patients with relapsed or refractory multiple myeloma. Blood.

[222] Rosebeck S., Alonge M.M., Kandarpa M., Mayampurath A., Volchenboum S.L., Jasielec J.K., Dytfeld D., Maxwell S.P., Kraftson S.J., McCauley D. (2016). Synergistic Myeloma Cell Death via Novel Intracellular Activation of Caspase-10–Dependent Apoptosis by Carfilzomib and Selinexor. Mol. Cancer Ther..

[223] Turner J.G., Kashyap T., Dawson J.L., Gomez J., Bauer A.A., Grant S., Dai Y., Shain K.H., Meads M., Landesman Y. (2016). XPO1 inhibitor combination therapy with bortezomib or carfilzomib induces nuclear localization of IκBα and overcomes acquired proteasome inhibitor resistance in human multiple myeloma. Oncotarget.

[224] Van Andel H., Kocemba K.A., Spaargaren M., Pals S.T. (2019). Aberrant Wnt signaling in multiple myeloma: Molecular mechanisms and targeting options. Leukemia.

[225] Cao Z., Liao Q., Su M., Huang K., Jin J., Cao D. (2019). AKT and ERK dual inhibitors: The way forward?. Cancer Lett..

[226] Auclair D., Lonial S., Anderson K.C., Kumar S.K. (2019). Precision medicine in multiple myeloma: Are we there yet?. Expert Rev. Precis. Med. Drug Dev..

[227] Broad Institute https://www.broadinstitute.org/cancer/cancer-dependency-map.

[228] Woodcock J., LaVange L.M. (2017). Master Protocols to Study Multiple Therapies, Multiple Diseases, or Both. N. Engl. J. Med..

[229] Mogollón P., Díaz-Tejedor A., Algarín E.M., Paíno T., Garayoa M., Ocio E.M. (2019). Biological Background of Resistance to Current Standards of Care in Multiple Myeloma. Cells.

[230] Laganà A., Beno I., Melnekoff D., Leshchenko V., Madduri D., Ramdas D., Sanchez L., Niglio S., Perumal D., Kidd B.A. (2018). Precision Medicine for Relapsed Multiple Myeloma on the Basis of an Integrative Multiomics Approach. JCO Precis. Oncol..

